# The *Mycobacterium tuberculosis* Ku C-terminus is a multi-purpose arm for binding DNA and LigD and stimulating ligation

**DOI:** 10.1093/nar/gkac906

**Published:** 2022-10-17

**Authors:** Dana J Sowa, Monica M Warner, Andriana Tetenych, Lucas Koechlin, Pardis Balari, Jose Pablo Rascon Perez, Cody Caba, Sara N Andres

**Affiliations:** Biochemistry and Biomedical Sciences, McMaster University, Hamilton, Ontario L8S 4K1, Canada; Michael DeGroote Institute for Infectious Disease Research, McMaster University, Hamilton, Ontario L8S 4L8, Canada; Biochemistry and Biomedical Sciences, McMaster University, Hamilton, Ontario L8S 4K1, Canada; Michael DeGroote Institute for Infectious Disease Research, McMaster University, Hamilton, Ontario L8S 4L8, Canada; Biochemistry and Biomedical Sciences, McMaster University, Hamilton, Ontario L8S 4K1, Canada; Michael DeGroote Institute for Infectious Disease Research, McMaster University, Hamilton, Ontario L8S 4L8, Canada; Biochemistry and Biomedical Sciences, McMaster University, Hamilton, Ontario L8S 4K1, Canada; Michael DeGroote Institute for Infectious Disease Research, McMaster University, Hamilton, Ontario L8S 4L8, Canada; Biochemistry and Biomedical Sciences, McMaster University, Hamilton, Ontario L8S 4K1, Canada; Biochemistry and Biomedical Sciences, McMaster University, Hamilton, Ontario L8S 4K1, Canada; Biochemistry and Biomedical Sciences, McMaster University, Hamilton, Ontario L8S 4K1, Canada; Michael DeGroote Institute for Infectious Disease Research, McMaster University, Hamilton, Ontario L8S 4L8, Canada; Biochemistry and Biomedical Sciences, McMaster University, Hamilton, Ontario L8S 4K1, Canada; Michael DeGroote Institute for Infectious Disease Research, McMaster University, Hamilton, Ontario L8S 4L8, Canada

## Abstract

Bacterial non-homologous end joining requires the ligase, LigD and Ku. Ku finds the break site, recruits LigD, and then assists LigD to seal the phosphodiester backbone. Bacterial Ku contains a core domain conserved with eukaryotes but has a unique C-terminus that can be divided into a minimal C-terminal region that is conserved and an extended C-terminal region that varies in sequence and length between species. Here, we examine the role of *Mycobacterium tuberculosis* Ku C-terminal variants, where we removed either the extended or entire C-terminus to investigate the effects on Ku–DNA binding, rates of Ku-stimulated ligation, and binding affinity of a direct Ku–LigD interaction. We find that the extended C-terminus limits DNA binding and identify key amino acids that contribute to this effect through alanine-scanning mutagenesis. The minimal C-terminus is sufficient to stimulate ligation of double-stranded DNA, but the Ku core domain also contributes to stimulating ligation. We further show that wildtype Ku and the Ku core domain alone directly bind both ligase and polymerase domains of LigD. Our results suggest that Ku-stimulated ligation involves direct interactions between the Ku core domain and the LigD ligase domain, in addition to the extended Ku C-terminus and the LigD polymerase domain.

## INTRODUCTION

Bacteria that reside in low metabolic states over extended periods of time, like sporulation or stationary phase, would be challenged to repair DNA double-strand breaks (DSBs) by homologous recombination, which requires a DNA template (1). The discovery of a non-homologous end-joining pathway (NHEJ) in bacteria, which does not use a DNA template for repair, provided a solution to DSB repair when homologous recombination is not possible. NHEJ was first predicted to exist and then characterized in multiple bacterial species, including *Bacillus subtilis*,*Mycobacterium tuberculosis* and *Pseudomonas aeruginosa* (2–6), through homology to the eukaryotic NHEJ proteins. Bacterial NHEJ is a minimalist version of eukaryotic NHEJ, consisting primarily of a Ku protein for binding DNA and recruiting the ATP-dependent ligase, LigD (4,5,7–9), although additional proteins can be involved under certain circumstances (10,11).

Similar to NHEJ in eukaryotes, the DSB is recognized by Ku, which binds the DNA ends, recruiting LigD to repair the break (2,4,5). Single-molecule studies of Ku from *B. subtilis* show that Ku binds to and bridges a DSB (12,13), but also maintains lyase activity in *P. aeruginosa* and *B. subtilis* (14), a function conserved with human Ku (15). Critically, Ku recruits the ATP-dependent ligase, LigD. The multi-functional LigD contains a polymerase, ligase, and a phosphoesterase domain that is absent in some species, like *B. subtilis* (4,9,16–18). The polymerase domain permits DSB repair without a template, as it can carry out non-templated or templated nucleotide addition, including the addition of ribonucleotides (19,20). The phosphoesterase domain has 3′-ribonuclease activity and ultimately converts a 3′-phosphate DNA end to a 3′-hydroxyl. These processing steps are necessary so that the chemistry of the ligation reaction can proceed by the ligase domain, in the presence of 3′-hydroxyl and 5′-phosphate DNA ends and ATP (6,17).

**Figure 1.**
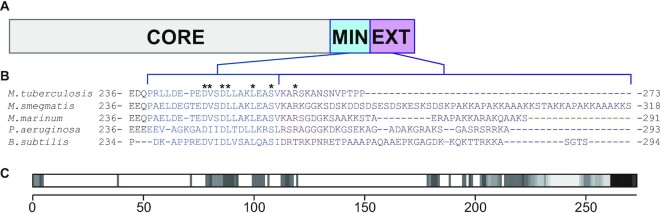
Ku contains a C-terminal domain unique to prokaryotes. (**A**) Domain arrangement for bacterial Ku. Ku contains an N-terminal core (CORE) domain, and a C-terminal domain that is composed of a minimal (MIN) and extended (EXT) region (13). Brackets outline the MIN and EXT regions in (**B**). Sequence alignment of the Ku C-terminus in bacteria. Sequences are from *M. tuberculosis* (UniProt ID: P9WKD9), *M. smegmatis* (UniProt ID: A0A0D6IZT4), *Mycobacterium marinum* (UniProt ID: A0A1C9J7K9), *P. aeruginosa* (UniProt ID: Q9I1W5) and *B. subtilis* (UniProt ID: O34859). Sequence alignment was generated by Clustal Omega (38). The minimal C-terminal region is highlighted in blue and surrounded by blue brackets from (A), while the extended C-terminal region is highlighted in purple and surrounded by purple brackets from (A). Conserved residues targeted for alanine-scanning mutagenesis are indicated by an asterisk. (**C**) Structural disorder prediction for *M. tuberculosis* Ku, as calculated by D^2^P^2^ (35). Darker bars indicate a higher consensus of disorder predicted for that sequence.

While function is generally conserved, there are some unique sequence variations in Ku between bacterial species. An in-depth *in silico* comparative sequence analysis identified 528 bacterial genomes containing a unique *ku* gene (13). This analysis delineated regions within Ku, based on sequence, of a conserved core domain, followed by a C-terminus sub-divided into a conserved minimal and variable extended region (2,13) (Figure 1A). The core domain is conserved throughout bacteria with the *ku* gene, and maintains homology with human Ku70 (13). The structure of the human Ku70/80 heterodimer forms a ring through which DNA can thread (21). Small-angle X-ray scattering (SAXS) and *in silico* models of *B. subtilis* Ku suggest the same ring-shaped core is formed, but bacteria possess a unique C-terminus not found in its eukaryotic homologue (12,13). From the sequence analysis, the minimal C-terminal region is conserved amongst bacteria, while the extended region varies in sequence and length, ranging from 1 to 155 amino acids, and is characteristically basic in nature (13). The structure of this C-terminus from SAXS modelling suggests a flexible, unstructured region (13), while *in silico* modelling suggests the C-terminus forms an alpha helix, with an additional disordered region (12,22,23).

The Ku C-terminus has many functional roles in NHEJ. While the Ku core domain from *B. subtilis* can synapse DNA ends, the Ku C-terminus further stabilizes this synapsis and stimulates LigD ligation of blunt-ended double-stranded DNA (dsDNA) (12). It is the minimal C-terminus, though, that is sufficient to interact with LigD, a feature shared with Ku from *P. aeruginosa* (12,13,20). Moving down the Ku protein, the Ku extended C-terminal region from both *B. subtilis* and *Mycobacterium smegmatis*, permits binding to supercoiled DNA (13,24). This extended C-terminal region also limits translocation on linear dsDNA for *B. subtilis* Ku and plays a role in stimulating ligation (12,13). How do these functions compare to Ku proteins that have significantly different extended C-termini? *M. tuberculosis* Ku contains only a 14 amino acid long extended C-terminal region, compared to 40 amino acids in *B. subtilis* and >50 amino acids in *M. smegmatis* (13) (Figure 1B). Functionally, *M. tuberculosis* Ku binds a minimum of ∼30 bp dsDNA, interacts with LigD through the LigD polymerase domain, and stimulates ligation of a sticky-ended dsDNA substrate (4,17). However, given the importance of the minimal and extended C-termini of Ku in other species, how does the C-terminus of *M. tuberculosis* Ku impact DNA binding, interaction with LigD, and ligation of dsDNA that mimics a DSB?

Here, we take a quantitative approach to examine the effects of the core domain, the minimal C- and the extended C-terminal regions of Ku from *M. tuberculosis* to gain greater insight into the mechanism of Ku-stimulated ligation. Interestingly, we find that the Ku C-terminus limits DNA binding and identify specific amino acids that contribute to DNA binding regulation. Furthermore, we show that even though the extended C-terminus is shorter compared to other homologs, it is critical to interact with LigD, while the minimal C-terminus is sufficient to stimulate ligation of blunt and sticky-ended dsDNA substrates. Finally, we examine the ligation activity of the LigD ligase domain and find that the ligase domain can only ligate nicks on its own. Furthermore, while the extended Ku C-terminus stimulates nick-sealing of wildtype LigD, it does not stimulate nick-sealing by the ligase domain alone. Based on these results and our findings that Ku is also capable of binding the LigD polymerase domain, we propose a mechanism where both direct and allosteric interactions between Ku and LigD contribute to Ku-stimulated ligation.

## MATERIALS AND METHODS

### Plasmid cloning

Codon-optimized Ku (ID: P9WKD9) and LigD (ID: P9WNV3) sequences from *M. tuberculosis* were synthesized by Genewiz and cloned through ligation-independent cloning (LIC) (25) using *SspI* restriction endonuclease (NEB, R0132L) and pMCSG7 expression vectors with a TEV-cleavable, N-terminal 6xHis-tag (26) (Addgene). Oligonucleotide primers (Integrated DNA Technologies) are described in Supplementary Table S1. All truncations of Ku and LigD were created by LIC using pMCSG7 or by site-directed mutagenesis (27). All plasmids were verified using Sanger sequencing (Genewiz).

### Recombinant protein expression and purification

Expression plasmids for all Ku and LigD proteins were grown in Luria-Bertani (LB) broth at 37°C in *Escherichia coli* BL21 (DE3) codon plus (Invitrogen) with expression induced at an OD_600_ of ∼0.7 with 0.1 mM IPTG for 16 h at 16°C. Bacterial cells were harvested by centrifugation, resuspended in lysis buffer (50 mM Tris–HCl pH 8.0, 1 M NaCl, 2 mM β-mercaptoethanol, 10% (w/v) sucrose, 5% (v/v) glycerol, 0.1% (v/v) Triton X-100, 0.1% (v/v) NP-40, 0.5 mg/ml lysozyme), and lysed by sonication. Lysate was clarified by centrifugation and affinity purified by Ni-NTA IMAC resin (Bio-Rad). Ni-NTA resin was washed with Ni-NTA Wash Buffer (50 mM Tris–HCl pH 8.0, 400 mM NaCl, 10% (v/v) glycerol) and eluted in a stepwise gradient with 20, 40 and 400 mM imidazole in Ni-NTA wash buffer. Ku-containing elutions were further purified by anion exchange chromatography, using a 5 ml HiTrap Q HP column (GE Healthcare), equilibrated with Q buffer (20 mM Tris–HCl pH 8.0, 2 mM β-mercaptoethanol) and eluted over a gradient from 150 mM–1 M NaCl. LigD-containing elutions were purified by cation exchange chromatography, using a 5 ml HiTrap SP HP column (GE Healthcare) in the same Q buffer and salt gradient as Ku. Ku and LigD were further purified using size-exclusion chromatography (HiLoad 16/600 Superdex 200 pg, GE Healthcare) in S200 buffer (20 mM Tris–HCl pH 8.0, 400 mM NaCl, 10% (v/v) glycerol). Purified proteins were concentrated using a centrifugal concentrator (Milllipore) and were visualized by SDS-PAGE to assess purity. All Ku and LigD mutations were purified according to their wildtype conditions. SDS-PAGE of all purified proteins are available in Supplementary Figure S1. Proteins were stored at –80°C until further use. All protein concentrations throughout are expressed as protein concentrations for a monomeric state, including Ku.

### DNA substrate preparation

DNA substrates used in DNA binding and ligation assays were purchased as synthetic oligonucleotides from Integrated DNA Technologies (IDT). Oligonucleotide sequences and modifications can be found in Supplementary Table S2. Complementary DNA oligonucleotides were resuspended in milliQ H_2_O and equimolar concentrations of each were annealed at 95°C for 2 min and then cooled to 25°C over 45 min. DNA was then purified through ethanol precipitation and resuspended in milliQ H_2_O. Annealed and single-stranded DNA (ssDNA) substrates are shown in Supplementary Figure S2 on a 4–20% Native PAGE gel. The 10nt, 15nt ssDNA and 10 bp, 15 bp dsDNA were poorly resolved on the gel and thus not shown but had free energy (Δ*G*) values calculated by the IDT Oligo Analyzer (v3.1, Integrated DNA Technologies, Inc.) of –18.1 and –28.1 kcal/mol for the 10 and 15 bp dsDNA, respectively, indicating these substrates would readily anneal.

### Ku DNA binding assays

20 μl reactions contained EMSA buffer (50 mM Tris–HCl pH 8.0, 50 mM NaCl, 1 mM DTT, 5 mM MgCl_2_, 4% (v/v) glycerol, 0.025 mg/ml BSA) with 10 nM fluorescein-labelled DNA and 2-fold serial dilutions of protein, with maximum protein concentration as indicated in the respective figures. Reactions were incubated at 30°C for 20 min and resolved on an 8% native PAGE or 4–20% gradient PAGE gel. Electrophoresis was performed in 0.5X TBE buffer at room temperature (21°C). Experiments (*n* = 3 technical replicates at minimum) were imaged with an Amersham Typhoon Imager (GE Healthcare) and analysed with ImageJ by quantifying the intensity of free DNA as protein concentration increased (28). DNA binding curves were plotted as a function of the fraction of DNA bound compared to an unbound DNA control. Dissociation constants were obtained through calculation of specific binding with Hill slope in Prism v.9.0 (GraphPad). p-values were calculated by a two-tailed Welch's *t*-test in Prism v.9.0 (GraphPad).

### DNA bridging assays

DNA bridging assays for Ku_WT_, Ku_min_ and Ku_core_ were completed as previously described (29). Briefly, a biotinylated DNA substrate was created by PCR amplification from a pUC-19 plasmid with primers P1 and P2 (P1:5′Biotin-CACCTAGGAATTCCCCTGCCGCTTACCGG; P2: CACCTAGGAATTCGGGAACCGGAGCTGAATGAAG) to create a 1000 bp 5′-biotinylated DNA substrate. A 40 bp 5′-fluorescein labelled DNA substrate (used in DNA binding assays, Supplementary Table S2), was used as the DNA bridging partner for the biotinylated DNA substrate. 40 μl reactions contained DNA bridging buffer A (20 mM Tris–HCl, pH 7.5, 50 mM KCl, 5 mM EDTA, 1 mM DTT, 5% (v/v) glycerol and 40 ug/ml BSA), 200 ng of fluorescently labelled DNA, 200 ng of biotinylated DNA, and 0 or 10 μM of Ku_WT_, Ku_min_ or Ku_core_. Reactions were left to incubate at room temperature with gentle nutation for 30 min with 10 μl of Streptavidin Sepharose High Performance resin (GE Healthcare). Following incubation, 3 × 20 μl washes were completed using DNA bridging buffer B (20 mM Tris–HCl pH 7.5, 50 mM KCl, 5 mM EDTA, 1 mM DTT, 5% (v/v) glycerol). Reactions were deproteinated at 65°C using a mixture of 10 μg/μl of Proteinase K and 0.5% SDS and visualized on a 4–20% non-denaturing PAGE in 1× TBE. Electrophoresis was conducted at 200 V for 40 min. Products were visualized using the Amersham Typhoon Imager (GE Healthcare), and DNA bridging was quantified using ImageJ. (28).

### dsDNA end ligation assay

50 μl reaction mixtures containing ligation buffer (50 mM HEPES, pH 7.5, 5 mM MgCl_2_, 5 mM DTT, 1 mM ATP and 10 U/ml of pyrophosphatase) with 20 ng of pUC19 plasmid, linearised with either *KpnI* (ThermoFisher) or *SmaI* (NEB), were incubated with 0.5 μM of LigD, in the presence or absence of 1 μM of Ku_WT_, Ku_min_, or Ku_core_, at 37°C for 30 min. Reactions were quenched at 0, 5, 10, 15, 20, 25 and 30 min with 100 μl of Biomol Green reagent (Enzo Life Sciences). The Biomol Green reagent allows for a coupled enzymatic reaction, where inorganic pyrophosphate released by ligation is converted to phosphate by pyrophosphatase. The release of phosphate results in a colorimetric readout that increases in a linear proportion with the phosphate concentration. These colorimetric changes can be observed through measuring the OD at 620 nm by a Synergy Neo2 plate reader (Biotek). To establish that the release of pyrophosphate by LigD was responsible for observed changes in the rate of the reaction (rate limiting step), and not the release of phosphate by pyrophosphatase, we mixed 2-fold serial dilutions of LigD with our master mix and plotted LigD concentration versus OD_620_ (Supplementary Figure S3A). The resulting linear relationship indicates that release of pyrophosphate by LigD is the rate limiting step of the coupled reaction.

DNA ligation results were plotted as a function of the phosphate released (nmol), as determined by the standard curve in Supplementary Figure S3B, versus time (min). Slopes from these plots were calculated by simple linear regression in Prism v. 9.0 (GraphPad), which corresponded to the rate of the reaction. *p*-values were calculated by a two-tailed Welch's *t*-test in Prism v. 9.0 (GraphPad).

### Nicked ligation assay

The ligation assay was adapted from a previously published protocol (30–32). Briefly, 20 μl ligation reaction mixtures containing 50 mM HEPES pH 8.0, 5 mM DTT, 5 mM MgCl_2_, 250 μM ATP with 50 nM of 3′-fluorescein labelled, centrally nicked duplex DNA substrates were incubated with 1 μM Ku and 0.5 μM LigD at 37°C for 15 min, quenching at time points 0, 0.5, 1, 2.5, 5, 7.5, 10, 12.5, 15 min with 98% (v/v) formamide and 200 mM EDTA. The control reaction containing only LigD and DNA was incubated for 15 min. The ligation products (*n* = 3 technical replicates) were resolved on a 20% denaturing–urea PAGE gel at 200 V for 45 min and were visualized by an Amersham Typhoon Imager (GE Healthcare). Ligation products were quantified using ImageJ (28). DNA ligation results were plotted as a fraction of DNA product ligated compared to an unligated DNA control. Slopes were calculated by simple linear regression in Prism v.9.0 (GraphPad). Rate was obtained through the calculation of the concentration of product formed over time. *p*-values were calculated by a two-tailed Welch's *t*-test in Prism v.9.0 (GraphPad).

### Ku biotinylation for biolayer interferometry

Ku was biotinylated following the protocols of the EZ-Link™ NHS-LC-Biotin kit (ThermoScientific, catalogue #21343) (33). Unreacted biotin was removed by size-exclusion chromatography (Superdex 75 10/300 GL, GE Healthcare) and biotinylated protein eluted in S75 buffer (20 mM HEPES pH 8.0, 400 mM NaCl). Protein was concentrated using a centrifugal concentrator (Millipore) prior to use. A list of the biotinylated sites on Ku_WT_, Ku_min_ and Ku_core_ is available in Supplementary Table S3. Multiple biotinylation sites were used to allow for multiple conformations of Ku when fixed to the BLI sensor, so that all potential LigD binding sites were accessible. Biotinylation sites were identified by mass spectrometry performed by SPARC BioCentre Molecular Analysis, The Hospital for Sick Children, Toronto, Canada.

### Biolayer interferometry (BLI)

BLI analyses were performed using an Octet RED96 system (ForteBio). Assays were performed in a black 96-well plate with a flat bottom at 37°C and shaking at 1000 rpm. The total working volume for each well was 200 μl. Prior to each assay, streptavidin (SA) biosensors (ForteBio) were soaked in 1× kinetics buffer (0.1% (w/v) BSA, 0.02% (v/v) Tween-20, 0.05% (w/v) sodium azide in PBS) for 10 min, followed by equilibration in 1× kinetics buffer for 60 s. Following equilibration, the SA biosensors were loaded with 25 μg/ml biotinylated Ku protein in 1× kinetics buffer for 120 s, followed by another 60 s equilibration period in 1× kinetics buffer. Association of 12.5 μg/ml LigD to immobilized Ku was performed for 300 s. Dissociation was carried out in 1× kinetics buffer for 300 s. *n* = 4 technical replicates were completed for Ku_WT_, Ku_core_ and Ku_min_ interactions while *n* = 3 technical replicates were completed for interactions with Ku proteins containing single point mutations. A simple kinetics assay was completed using the Octet data acquisition software v. 9.0.0.37 (ForteBio). Data was analysed using the Octet data analysis software v. 9.0.0.12 (ForteBio). Data were subtracted from a reference sensor and were fitted using a grouped global fit, which provided *K*_D_ values for the interaction between Ku and LigD proteins tested.

### LigD DNA binding by fluorescence polarization

30 μl reactions containing fluorescence polarization (FP) buffer (10 mM Tris–HCl buffer pH 8, 10 mM NaCl, 10% (v/v) glycerol and 1 mM TCEP) with 50 nM fluorescein-labelled DNA (10, 15, 20, 30 and 40 bp) and 2-fold serial dilutions of LigD were placed in a 384-well black-bottomed microplate. Reactions were incubated at 30°C for 20 min, before polarization was measured by a Synergy Neo2 plate reader (Biotek). The change in fluorescence polarization was measured and plotted as a function of protein concentration. Dissociation constants (*K*_D_) were calculated using Prism v.9.0 (GraphPad).

### Ternary complex electrophoretic mobility shift assays

20 μl reaction mixtures contained EMSA buffer (20 mM Tris–HCl pH 8.0, 5 mM MgCl_2_, 5% glycerol), 10 nM fluorescein-labelled 40 bp DNA and/or 1 μM LigD and/or 10 μM of Ku_WT_, Ku_min_ or Ku_core_. Reactions were incubated at 37°C for 20 min before addition of 4 μl of 50% glycerol for loading on a 6% non-denaturing gel in 0.5× TBE. Reactions were visualized using the Amersham Typhoon Imager (GE Healthcare).

### Size-exclusion chromatography coupled to multi-angle light scattering (SEC-MALS)

Proteins of interest were diluted in SEC-MALS Buffer (50 mM HEPES pH 8.0, 1 mM EDTA and 200 mM NaCl) to a final concentration of 50 μM and centrifuged in a refrigerated microcentrifuge at 21 000 × g for 10 min to remove any aggregated protein. The supernatant was loaded onto a Superdex 200 Increase 10/300 GL (GE Healthcare) on an AKTA Pure FPLC system (GE Healthcare) with MALS being conducted using a MiniDAWN and Optilab system (Wyatt Technology). Data was collected and analysed using the Astra software, version 7.3.1.9 (Wyatt Technology) and plotted in Prism v.9.0 (GraphPad).

## RESULTS

### The Ku C-terminus limits DNA binding

In most bacterial Ku homologs, the minimal C-terminus is well conserved, while the extended C-terminus varies widely (13) (Figure 1B). Compared to *B. subtilis* and *M. smegmatis*, *M. tuberculosis* Ku has an extended C-terminus that is 43 amino acids shorter than Ku in *M. smegmatis*, although the overall basic nature is maintained, with *M. tuberculosis* and *M. smegmatis* having a pI of 11.2 and 10.1 respectively (calculated by Compute pI/MW (34)). The entire C-terminus of *M. tuberculosis* Ku is also predicted by D^2^P^2^ to be intrinsically disordered (35) (Figure 1C), which is similar to models of *B. subtilis* Ku (12,13). However, *in silico* predictions of Ku by AlphaFold and ColabFold, suggest some structure within the *M. tuberculosis* Ku C-terminus (22,23,36). We cloned, expressed, and purified three Ku proteins based on the alignment in Figure 1B: (i) wildtype Ku (Ku_WT_), (ii) Ku_min_, where the extended C-terminus is removed and only the minimal C-terminal region remains and (iii) Ku_core_, where the entire C-terminus is removed, leaving behind the conserved core domain. We used these proteins to investigate how the variable extended C-terminus affects *M. tuberculosis* Ku function, compared to known functions of bacterial Ku homologues with > 14 amino acids in the extended C-terminus. Size-exclusion chromatography, coupled to multi-angle light scattering (SEC-MALS) confirmed a single oligomeric species for each protein purified, and that the Ku homodimer is maintained for Ku_WT_, Ku_min_ and Ku_core_ (Supplementary Figure S4 and Supplementary Table S4).

**Figure 2.**
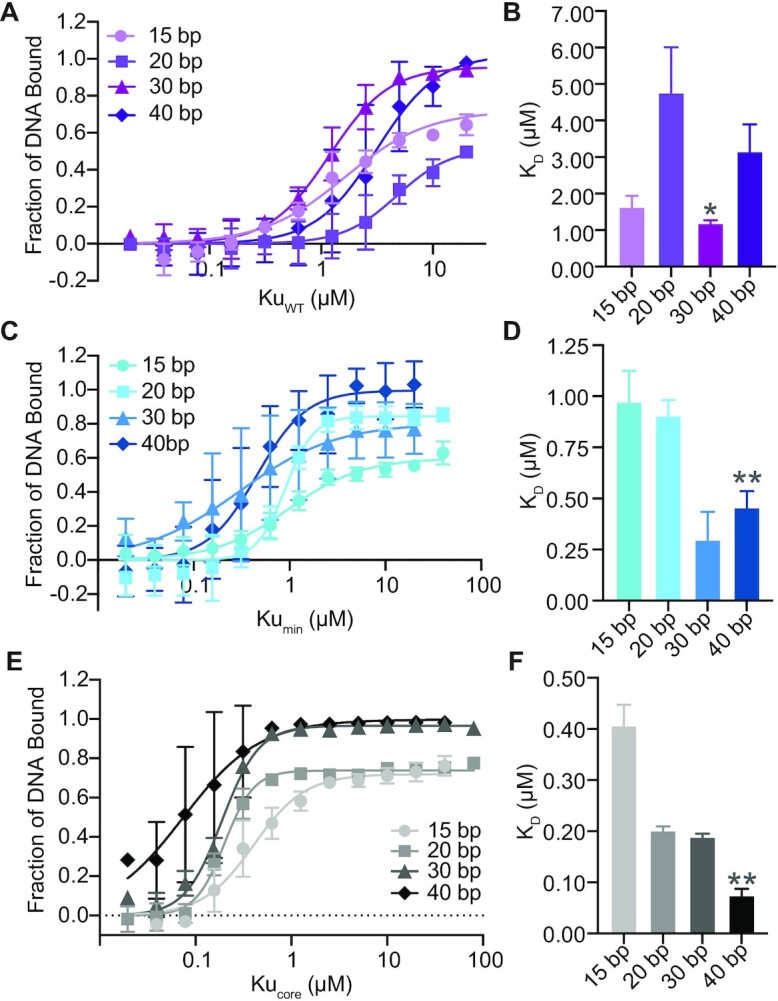
The Ku C-terminus limits DNA binding. (**A**) DNA binding curves for Ku_WT_ with 15, 20, 30 and 40 bp blunt-ended dsDNA substrates. Data were plotted as a fraction of DNA bound. (**B**) Apparent dissociation constants (*K*_D_) of Ku_WT_ binding to 15, 20, 30 and 40 bp dsDNA, calculated from (A). (**C**) DNA binding curves for Ku_min_ with 15, 20, 30 and 40 bp blunt-ended dsDNA substrates. Data were plotted as a fraction of DNA bound. (**D**) Apparent dissociation constants (*K*_D_) of Ku_min_ binding to 15, 20, 30 and 40 bp dsDNA, calculated from (C). (**E**) DNA binding curves for Ku_core_ with 15, 20, 30 and 40 bp blunt-ended dsDNA substrates. Data were plotted as a fraction of DNA bound. (**F**) Apparent dissociation constants (*K*_D_) of Ku_core_ binding to 15, 20, 30 and 40 bp dsDNA, calculated from (E). All data are plotted as mean ± SEM. *n* = 3 technical replicates. **p < 0.05 and *p< 0.1 (two-tailed *t*-test) in comparison to *K*_D_ for Ku_WT_ binding 40 bp dsDNA in (A).

Previous reports indicate that Ku from *M. tuberculosis* binds as little as 30 bp of DNA (4). We wanted to quantify how the Ku C-terminus affects this DNA interaction. We began by testing a variety of blunt-ended, fluorescently-labelled, dsDNA substrates ranging from 10 to 40 bp, using a standard electrophoretic mobility shift assay (EMSA) (Supplementary Figures S5–S7). None of the Ku proteins bound a 10 bp substrate (data not shown). Ku_WT_ bound everything from 15 to 40 bp, however, a higher fraction of DNA bound was observed with the longer DNA substrates of 30 and 40 bp, even though the apparent *K*_D_ was similar for 15 and 30 bp, at 1.63 ± 0.30 μM and 1.18 ± 0.09 μM, respectively. Therefore Ku_WT_ does have a smaller footprint of 15 bp compared to previous reports of at least a 30 bp footprint for *M. tuberculosis* Ku, although longer DNA likely provides a more stable complex (4) (Figure 2A and B). The Ku–DNA binding affinity greatly increases, though, as the C-terminus is removed. Ku_min_ binds 15–40 bp of DNA, with a 6-fold increase in affinity for the 40 bp substrate (*K*_D_ = 0.46 ± 0.08 μM) compared to wildtype Ku (Figure 2C and D), while the Ku_core_ binds all DNA substrates more tightly than Ku_WT_ and Ku_min_, with a >40-fold increase in affinity for the 40 bp DNA (*K*_D_ = 0.07 ± 0.01 μM) compared to Ku_WT_ (Figure 2E and F). Hill coefficients for all Ku proteins binding DNA of 20 bp or longer was >1, indicating cooperative binding. A full list of apparent *K*_D_ values for all DNA substrates and proteins tested is available in Supplementary Table S5. As the C-terminus of Ku is removed, DNA binding affinity increases, with both the minimal and extended C-terminus reducing protein–DNA affinity. These results indicate that the core domain, conserved with eukaryotes, remains the primary DNA binding site, while the C-terminus limits the high affinity Ku–DNA binding observed when only Ku_core_ is present.

We further wanted to pinpoint if the changes in DNA binding affinity could be attributed to the conserved amino acids throughout the C-terminus. We carried out alanine-scanning mutagenesis on these amino acids (Figure 1B). To confirm no major conformational changes were induced by these mutations, SEC-MALS analyses on all Ku point mutations, and the previous Ku truncations, indicated that every Ku mutant maintained the same elution volume and molecular weights corresponding to a dimer, except for Ku L255A (Supplementary Figure S4 and Supplementary Table S4). Ku L255A eluted at a lower volume than the other proteins, indicating that this point mutation induced a conformational change in the protein that likely affected its function. Therefore, L255A was removed from any further analysis.

**Figure 3.**
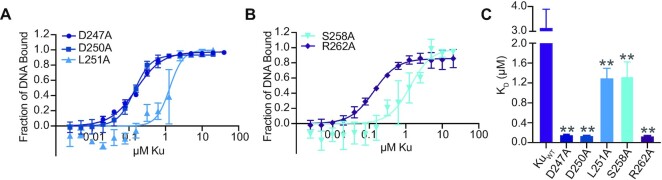
Conserved residues in the Ku C-terminus regulate DNA binding. (**A**) DNA binding curves for Ku D247A, D250A, and L251A binding a blunt-ended 40 bp dsDNA substrate. (**B**) DNA binding curves for Ku S258A and R262A binding to a blunt-ended 40 bp dsDNA substrate. (**C**) Apparent dissociation constants (*K*_D_) of Ku D247A, D250A, L251A, S258A and R262A binding to 40 bp dsDNA. All data are plotted as mean ± SEM. *n* = 3 technical replicates, except for Ku S258A, where *n* = 4 technical replicates. **p < 0.05 (two-tailed *t*-test) in comparison to Ku_WT_, which is included from Figure 2A as a reference.

We quantified binding affinity of the Ku point mutants on the 40 bp dsDNA used previously. Ku V248A was not included in testing, as it did not express. Interestingly, each purified Ku mutant had a statistically significant change in apparent *K*_D_ compared to Ku_WT_ alone, with an overall trend in increased affinity (Figure 3A–C, Supplementary Figure S8), with co-operativity maintained (Hill coefficient > 1). Ku mutants D247A (K_D_ = 0.16 ± 0.01 μM) and D250A (*K*_D_ = 0.14 ± 0.01 μM) in the minimal C-terminus and R262A (*K*_D_ = 0.14 ± 0.01 μM) in the extended C-terminus increased DNA binding affinity ∼20-fold higher compared to Ku_WT_. Ku S258A (*K*_D_ = 1.34 ± 0.29 μM) in the minimal C-terminus had an increased DNA binding affinity compared to Ku_WT_, although not as pronounced as the other Ku point mutants. Overall, these results suggest that in the wildtype protein, D247, D250, S258 and R262 from the C-terminus are involved in limiting binding of DNA to Ku. Surprisingly, the minor change of a conserved leucine to alanine in the minimal Ku C-terminus resulted in a moderately reduced apparent dissociation constant compared to Ku_WT_ (L251A, *K*_D_ = 1.30 ± 0.19 μM). Therefore, in wildtype Ku, L251 also contributes to regulating Ku–DNA binding.

### The Ku extended C-terminus promotes efficient DNA bridging

**Figure 4.**
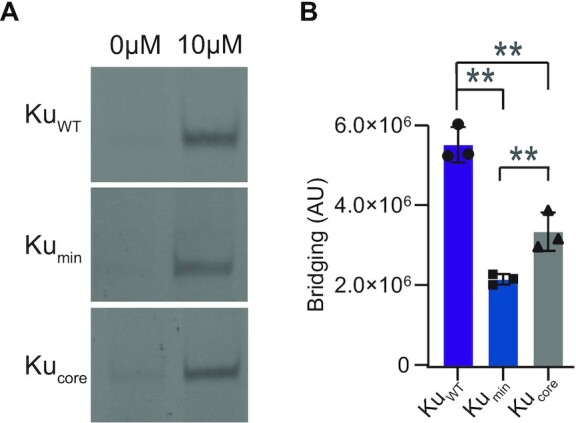
The Ku extended C-terminus bridges DNA efficiently. (**A**) Bridging was tested with 10 μM of protein, including Ku_WT_, Ku_min_ and Ku_core_ and visualized on a 20% non-denaturing PAGE. (**B**) Quantification of DNA bridged by 10 μM of Ku_WT_, Ku_min_ and Ku_core_. DNA bridging was quantified using densitometry, and data were plotted as the mean ± standard deviation (SD), with replicate data points included. *n* = 3 technical replicates. **p < 0.05 (two-tailed *t*-test).

Both transmission electron microscopy imaging and a DNA bridging study show that the extended C-terminus of *B. subtilis* Ku is needed to efficiently bridge between DNA molecules (13), while single-molecule studies with *B. subtilis* Ku show that both wildtype and Ku_core_ can synapse DNA ends (12). We wanted to examine whether *M. tuberculosis* Ku was able to bridge between two DNA molecules, and whether the Ku C-terminus affected bridging (Figure 4). Using a previously published bridging assay (29), we found that loss of the Ku C-terminus led to a reduction in DNA bridging (Figure 4). Quantification of DNA bridging showed a greater reduction in bridging with loss of the extended C-terminus (Ku_min_), compared to removal of the entire C-terminus (Ku_core_). Therefore, while *M. tuberculosis* Ku_core_ can bridge between two DNA molecules, as seen with *B. subtilis* Ku in single-molecule studies, the Ku extended C-terminus is required for efficient bridging, even though it limits DNA binding affinity.

### The Ku C-terminus stimulates LigD ligation and nick-sealing

**Figure 5.**
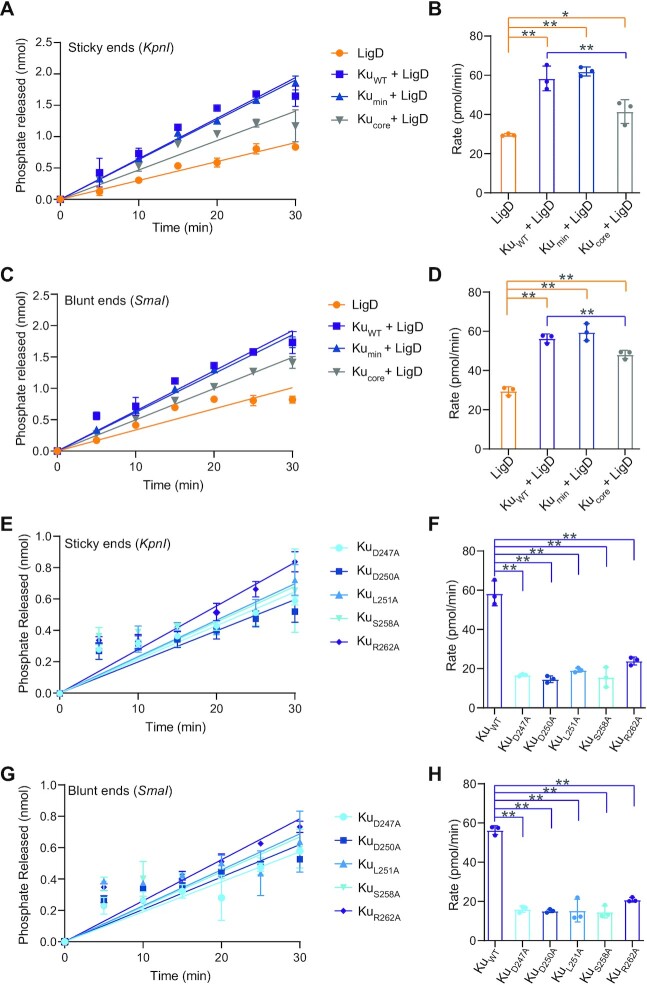
The Ku C-terminus and core domain stimulates LigD ligation of dsDNA. The DNA substrate used was a plasmid linearised with either *KpnI* to produce 4nt sticky ends, or *SmaI* to produce blunt ends. (**A**) Ligation of DNA with 4nt sticky ends, by LigD alone, or in the presence of Ku_WT_, Ku_min_ and Ku_core_. Data were plotted as a function of phosphate released (nmol) over time (min). Slopes were calculated by simple linear regression in Prism v.9.0 (GraphPad). (**B**) Rates for ligation of DNA with 4nt sticky ends, by LigD alone, or stimulated by Ku_WT_, Ku_min_ or Ku_core_. (**C**) Ligation of a blunt ended dsDNA substrate by LigD alone, or in the presence of Ku_WT_, Ku_min_ and Ku_core_. Data were plotted as a function of phosphate released (nmol) over time (min). Slopes were calculated by simple linear regression in Prism v.9.0 (GraphPad). (**D**) Rates for ligation of a blunt ended dsDNA substrate by LigD alone, or stimulated by Ku_WT_, Ku_min_, or Ku_core_. (**E**) Ligation of DNA with 4nt sticky ends by LigD alone, or in the presence of Ku D247A, D250A, L251A, S258A and R262A. Data were plotted as a function of phosphate released (nmol) over time (min). Slopes were calculated by simple linear regression in Prism v.9.0 (GraphPad). (**F**) Rates for ligation of DNA with 4nt sticky ends, by LigD alone, or stimulated by Ku D247A, D250A, L251A, S258A or R262A. (**G**) Ligation of a blunt ended dsDNA substrate by LigD alone, or in the presence of Ku D247A, D250A, L251A, S258A and R262A. Data were plotted as a function of phosphate released (nmol) over time (min). Slopes were calculated by simple linear regression in Prism v.9.0 (GraphPad). (**H**) Rates for ligation of a blunt ended dsDNA substrate by LigD alone, or stimulated by Ku D247A, D250A, L251A, S258A or R262A. All data plotted are the mean ± SD with replicate data points included in (B, D, F and H). *n* = 3 technical replicates. **p< 0.05 and *p < 0.1 (two-tailed *t*-test) in comparison to LigD rate (orange lines) or Ku_WT_ with LigD rate (purple lines). Ku_WT_-stimulated ligation rates for the defined DNA ends are included in (F) and (H) as reference points.

In *B. subtilis*, the Ku C-terminus is required to stimulate ligation, with the extended C-terminus present having a greater effect. *B. subtilis* Ku C-termini also aid in binding and bridging DNA (13), suggesting that DNA binding by the C-terminus would contribute to stimulating ligation by LigD. Given our findings that the Ku C-terminus in *M. tuberculosis* impedes high affinity DNA binding, we wanted to investigate how the Ku C-terminus of *M. tuberculosis* impacts LigD ligation. We examined whether Ku could stimulate ligation of dsDNA. To mimic a DSB, we linearised a pUC19 plasmid to produce either complementary 4-nt overhangs (sticky ends) or blunt ends. We then quantified the rate through a coupled enzymatic reaction, as described in the methods. Ligation rates were almost identical for both complementary and blunt DNA ends (Supplementary Tables S6 and S7). LigD alone can ligate the DSBs at a rate of ∼30 pmol/min, while addition of Ku_WT_ or Ku_min_ both doubled ligation rates compared to LigD alone (Ku_WT_, sticky = 58.4 ± 6.3; blunt = 56.4 ± 2.3 pmol/min; Ku_min_, sticky = 61.9 ± 2.3; blunt = 59.6 ± 4.4 pmol/min) (Figure 5A–D). Interestingly, Ku_core_ also increased ligation, but only by an additional ∼10pmol/min compared to LigD alone. Therefore, the minimal C-terminus, which is conserved between bacterial species, is the main Ku C-terminal feature required to stimulate ligation of a DSB *in vitro*, which is similar to results from *B. subtilis* (13).

We also looked at the effects of the single point mutations of the conserved Ku C-terminal residues on LigD ligation of dsDNA (Figure 5E–H). All Ku point mutations failed to stimulate ligation of the DSB (sticky ends: D250A = 14.6 ± 1.7 to R262A = 23.9 ± 2.0 pmol/min; see Supplementary Tables S6 and S7 for all rates), with rates similar to LigD alone (29.6 ± 0.6 pmol/min), which was surprising given that all Ku point mutations had higher DNA binding affinity than Ku_WT_.

**Figure 6.**
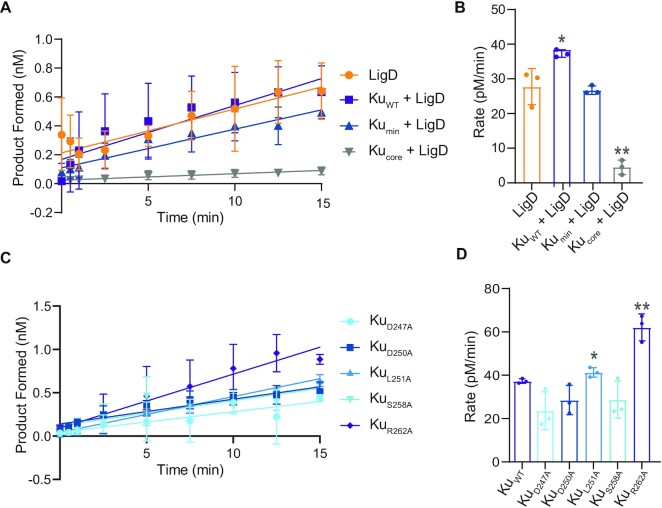
The extended Ku C-terminus stimulates LigD nick-sealing. (**A**) Ligation of a nicked DNA substrate by LigD alone, or in the presence of Ku_WT_, Ku_min_ and Ku_core_. Data were plotted as a function of the DNA product formed (nM) over time (min). Slopes were calculated by simple linear regression in Prism v.9.0 (GraphPad). (**B**) Rates for Ku-stimulated LigD nick-sealing in pM/min, by Ku_WT_, Ku_min_, Ku_core_ and LigD alone. (**C**) Ligation of a nicked DNA substrate by LigD in the presence of Ku point mutants. Data were plotted as a function of the DNA product formed (nM) over time (min). Slopes were calculated by simple linear regression in Prism v.9.0 (GraphPad). (**D**) Rates for Ku-stimulated LigD nick-sealing in pM/min, by Ku point mutants. Ku_WT_ from (B) is included for reference. All data are plotted as mean ± SD, with replicate data points included in (B) and (D). *n* = 3 technical replicates. **p < 0.05 and *p < 0.1 (two-tailed *t*-test) in comparison to LigD alone in (B).

We also examined nick-sealing rates by adapting a previously described assay (30–32), by measuring the product formed over time using a 36bp, fluorescently labelled dsDNA, with a centrally located nick. Alone, LigD can seal a nicked DNA substrate efficiently at a rate of 27.8 ± 4.1 pM/min (Figure 6A, B and Supplementary Table S8). Ku on its own cannot ligate a nick (Supplementary Figure S9), but when combined with LigD, the rate of ligation increases to 37.3 ± 1.3 pM/min, compared to LigD alone. Interestingly, removal of the extended C-terminus reduces nick-sealing rates similar to that of LigD alone (Ku_min_ + LigD, rate = 26.8 ± 1.3 pM/min), indicating that the extended C-terminus, which is variable between bacterial species, is part of the mechanism to stimulate ligation of a nick *in vitro*. Surprisingly, removing the entire Ku C-terminus severely inhibits LigD nick-sealing, with a rate of 4.5 ± 2.0 pM/min, suggesting that the Ku extended C-terminus is required to stimulate LigD nick-sealing.

We also looked at the effects of the single point mutations of the conserved Ku C-terminal residues on LigD nick-sealing (Figure 6C and D, Supplementary Figure S10). Contrary to our hypothesis that efficient DNA binding leads to higher nick-sealing rates, Ku point mutations that had higher DNA binding activity, have no effect, or have lower rates of nick-sealing ligation (D247A, D250A, S258A, rates in Supplementary Table S8), compared to Ku_WT_, suggesting these point mutations with higher DNA binding affinity are impeding LigD nick-sealing. However, there are two exceptions to these results. Ku L251A and Ku R262A, when combined with LigD, increased the rate of ligation to 41.3 ± 2.2 and 62.1 ± 6.2 pM/min, respectively, approximately doubling the nick-sealing rate of LigD alone and increasing the rate compared to ligation with Ku_WT_.

### The Ku extended C-terminus is critical for interaction with LigD

**Figure 7.**
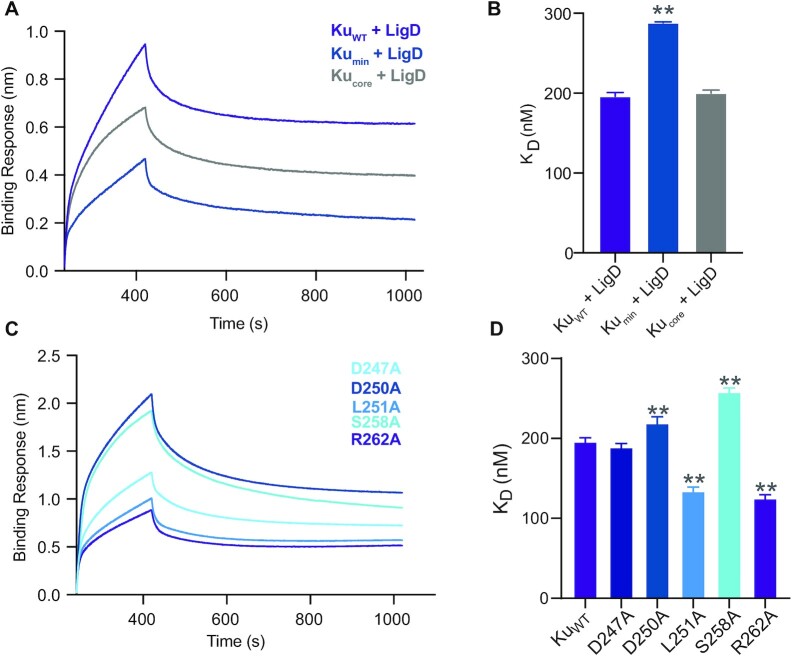
The Ku extended C-terminus mediates a direct, functional LigD interaction. (**A**) Association and dissociation curves of the LigD analyte with Ku proteins as ligands, over time, as determined by BLI. (**B**) Dissociation constants (*K*_D_) for LigD binding Ku_WT_, Ku_min_ or Ku_core_. Data are plotted as the mean ± SD for *n* = 4 technical replicates. (**C**) Association and dissociation curves of the LigD analyte with Ku proteins as ligands, over time, as determined by BLI. Ku proteins are the point mutations indicated. (**D**) Dissociation constants (*K*_D_) for LigD binding Ku D247A, D250A, L251A, S258A or R262A. Ku_WT_ from (B) is included for reference. Data are plotted as the mean ± SD for *n* = 3 technical replicates. **p < 0.05 (two-tailed *t*-test) in comparison to Ku_WT_ binding LigD in (B).

An additional factor that would contribute to stimulating LigD ligation is a direct protein-protein interaction between Ku and LigD. Studies using *B. subtilis* Ku and LigD found by gel filtration that the interaction between these proteins required the minimal Ku C-terminus (13). Using BLI, we examined if the same interactions were maintained by *M. tuberculosis* Ku (Figure 7). Beginning with Ku_WT_, Ku_min_ and Ku_core_ proteins as ligands, we found that all three proteins were capable of interaction with the analyte, LigD. The *K*_D_ between Ku_WT_ and LigD was 196 ± 5.0 nM (Figure 7A and B; Supplementary Table S9), while Ku_min_ had reduced affinity for LigD (*K*_D_ = 288 ± 1.4nM). Differing from results in other species, though, Ku_core_ (K_D_ = 200 ± 4.0 nM) had a similar affinity for LigD as Ku_WT_, suggesting that the Ku_core_ is the minimal domain required to interact with LigD, and is also capable of stimulating ligation of dsDNA, though not to the same extent as Ku_WT_.

Interestingly, the majority of the Ku point mutations changed the binding affinity between Ku and LigD when compared to Ku_WT_ (Figure 7C and D). Ku D247A did not affect the interaction (*K*_D_ = 189 ± 4.6 nM). Ku D250A and S258A, though, had reduced affinity for LigD (*K*_D_ = 219 ± 8.1 nM and *K*_D_ = 258 ± 5.0 nM, respectively). However, Ku L251A and R262A increased the affinity of Ku for LigD (*K*_D_ = 134 ± 5.1 nM and *K*_D_ = 125 ± 4.7 nM, respectively), complementing the similar increase in nick-sealing rate observed with these same two Ku mutants.

### A Ku–LigD–DNA complex forms independently of the Ku C-terminus

**Figure 8.**
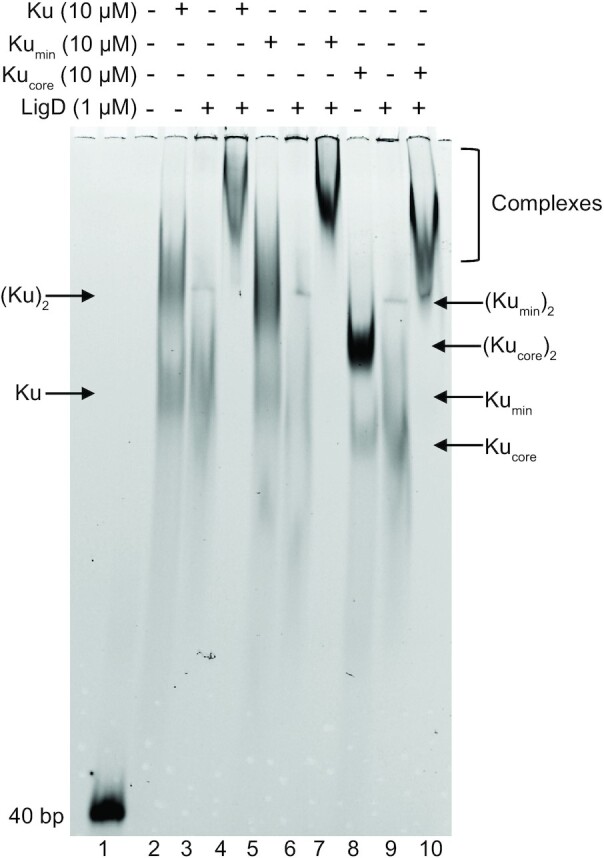
LigD forms ternary complexes with DNA and Ku_WT_, Ku_min_ or Ku_core_. 40 bp dsDNA was incubated with 10 μM of Ku_WT_, Ku_min_ or Ku_core_, and/or 1 μM LigD, as indicated above and analysed by EMSA. Ku–LigD–DNA complexes are identified based on band migration compared to the individual proteins alone. This image is a representative gel from three independent experiments, visualized by 6% non-denaturing PAGE.

Given that the Ku C-terminus plays an important role in stimulating LigD ligation, we wanted to examine the interplay of Ku and LigD on DNA. We ran a 40bp DNA substrate on a 4–20% Native PAGE gel and added Ku, LigD, or both proteins combined. For Ku_WT_, Ku_min_ or Ku_core_ we observed two separate gel shifts, indicating the DNA can accommodate two Ku dimers (Figure 8), as was also observed in the DNA binding assays (Supplementary Figures S5–S7). LigD can also bind 15–40 bp of DNA well, with an apparent *K*_D_ lower than Ku_WT_, but similar to Ku_min_ (LigD, 40 bp *K*_D_ = 0.35 ± 0.03 μM) (Supplementary Table S10, Supplementary Figure S11). LigD alone also produces two separate gel shifts (Figure 8). Some bands show a continual supershift, which can be indicative of multiple proteins binding DNA, multiple DNA binding sites on a protein, or bridging between DNA molecules (29). However, when LigD is combined with either Ku_WT_, Ku_min_ or Ku_core_, a super-shifted band is seen, with lower mobility than any of the proteins alone. These supershifts indicate that both Ku and LigD are present on the DNA and that removal of the C-terminus does not impair the formation of a ternary Ku–LigD–DNA complex.

### The Ku minimal C-terminus stimulates nick-sealing by the LigD ligase domain

The ligase domain (LIG) of LigD is the catalytic core for ligation activity, but the interaction between *M. tuberculosis* Ku and LigD has been proposed to reside in the polymerase domain (POL) (4). Therefore, we wanted to examine whether LIG alone was similarly stimulated by the extended Ku C-terminus, and if LIG and/or POL could interact with Ku.

**Figure 9.**
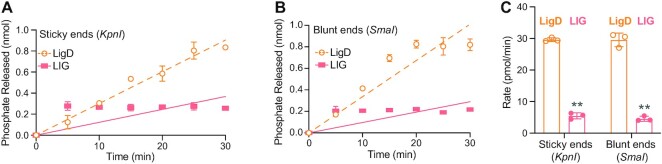
LigD LIG is inefficient at ligating dsDNA. The DNA substrate used was a plasmid linearised with either *KpnI* to produce 4nt sticky ends, or *SmaI* to produce blunt ends. (**A**) Ligation of DNA with 4nt sticky ends, by LigD and LIG. Data were plotted as a function of phosphate released (nmol) over time (min). Slopes were calculated by simple linear regression in Prism v.9.0 (GraphPad). (**B**) Ligation of a blunt ended dsDNA substrate by LigD and LIG. Data were plotted as a function of phosphate released (nmol) over time (min). Slopes were calculated by simple linear regression in Prism v.9.0 (GraphPad). (**C**) Ligation rates for LigD and LIG ligation of sticky and blunt ended DNA substrates in pmol/min. Data plotted are the mean ± SD for *n* = 3 technical replicates, with replicate data points included. **p < 0.05 (two-tailed *t*-test) in comparison to LigD.

We investigated the ability of LIG to ligate both sticky and blunt ends representative of a DSB, as described above with LigD. LIG on its own weakly ligated sticky and blunt ends, but only at a rate of about ∼5 pmol/min (blunt ends = 4.5 ± 1.4 pmol/min; sticky ends = 5.5 ± 1.8 pmol/min), 6-fold slower than LigD (Figure 9A–C), suggesting that additional domains of LigD are needed for faster ligation. We investigated if addition of Ku_WT_, Ku_min_ or Ku_core_ would stimulate LIG ligation of the DSBs, however the results were inconsistent (data not shown), further suggesting that the Ku extended C-terminus stimulates ligation by interacting with a domain of LigD other than LIG.

**Figure 10.**
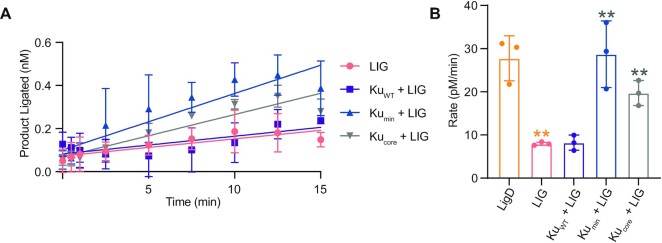
The extended Ku C-terminus hinders nick-sealing by the LigD ligase domain (LIG). (**A**) Ligation of a nicked DNA substrate by LIG alone, or with addition of Ku_WT_, Ku_min_ or Ku_core_. Data were plotted as a function of the DNA product formed (nM) over time (min). Slopes were calculated by simple linear regression in Prism v.9.0 (GraphPad). (**B**) Rates for nick-sealing by LIG or LIG in the presence of Ku_WT_, Ku_min_ or Ku_core_. LigD from Figure 6B is included for reference. Rates are plotted as mean ± SD for *n* = 3 technical replicates, with replicate data points included in (B). Double orange asterisks, **p < 0.05 (two-tailed *t*-test) in comparison to LigD nick-sealing rate in Figure 4B. Double grey asterisks, **p < 0.05 (two-tailed *t*-test) in comparison to LIG nick-sealing rate.

Therefore, we used the nick-sealing assay described earlier to test LIG activity in the presence and absence of our Ku_WT_, Ku_min_ and Ku_core_ proteins. We found that LIG is significantly slower at ligating a nicked substrate (rate = 7.9 ± 0.3 pM/min), compared to the wildtype LigD protein (rate = 27.8 ± 4.1 pM/min) (Figure 10 and Supplementary Table S8, Supplementary Figures S12). Interestingly, the addition of Ku_WT_ did not stimulate the nick-sealing rate of LIG (rates = 8.2 ± 1.8 pM/min), but loss of the extended or entire C-terminus (Ku_min_, Ku_core_) did, to levels like wildtype LigD with Ku_min_ + LIG (rate = 28.7 ± 7.7 pM/min) (Figure 10). These results suggest that the minimal C-terminus is sufficient to stimulate nick-sealing ligation through interaction with LIG, yet it is the extended C-terminus that is the smallest region needed to stimulate nick-sealing in wildtype LigD (Figure 6A and B).

**Figure 11.**
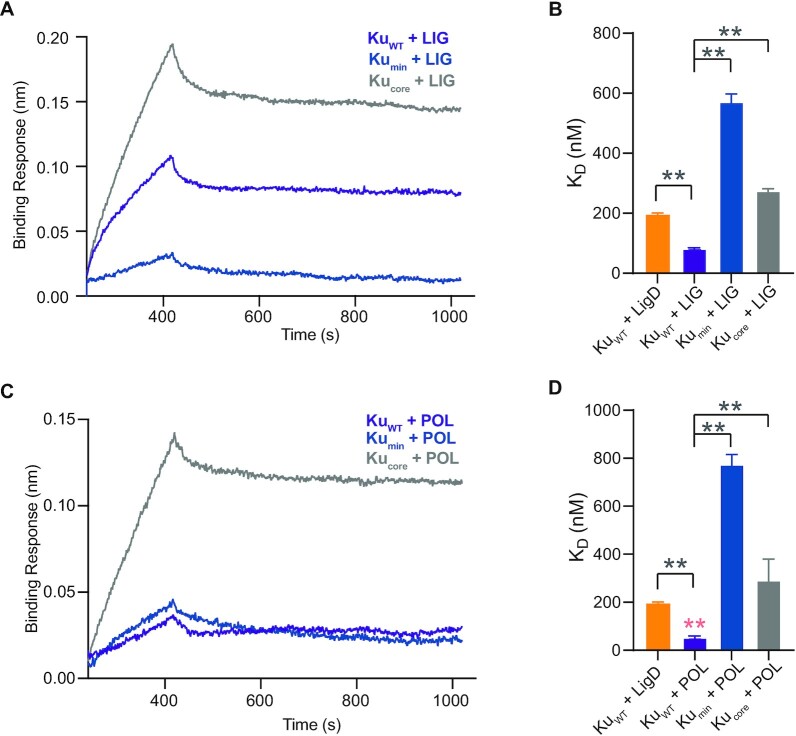
Ku_WT_ directly binds both the ligase and polymerase domains of LigD. (**A**) Association and dissociation curves of the LIG analyte with Ku proteins as ligands, over time, as determined by BLI. (**B**) Dissociation constants (*K*_D_) for LIG bound to Ku_WT_, Ku_min_ or Ku_core_. Data are plotted as the mean ± SD for *n* = 4 technical replicates. **p < 0.05 (two-tailed *t*-test). (**C**) Association and dissociation curves of the POL analyte with Ku proteins as ligands, over time, as determined by BLI. (**D**) Dissociation constants (*K*_D_) for POL bound to Ku_WT_, Ku_min_ or Ku_core_. Data are plotted as the mean ± SD for *n* = 4 technical replicates. Double pink asterisks, **p < 0.05 (two-tailed t-test) in comparison to *K*_D_ of Ku_WT_+ LIG in (B). Double grey asterisks, **p < 0.05 (two-tailed *t*-test). *K*_D_ of Ku_WT_+ LigD from Figure 7A are included for reference.

We next wanted to examine the interactions between our Ku proteins and the LIG and POL domains of LigD to see if changes in these interactions could explain our ligation results with LIG. Using BLI, with either Ku_WT_, Ku_min_ or Ku_core_ as ligand, and LIG or POL as analyte, we tested the affinity of the direct interaction between various protein combinations (Figure 11). Not surprisingly, Ku_WT_ bound both LIG and POL tighter (*K*_D_ = 81.4 ± 3.9 and 52.7 ± 7.3 nM, respectively, Supplementary Table S9) than Ku_min_ or Ku_core_ and tighter than LigD, confirming that the extended C-terminus is important for a direct interaction with the individual domains of LigD. Ku_WT_ though, bound POL tighter than LIG, confirming that the polymerase domain is likely the primary interaction site between Ku_WT_ and LigD. However, from these results, it is clear a direct interaction forms between Ku_WT_ and LIG, although it is likely not associated with ligation, based on the nick-sealing and ligation abilities of LIG in the presence of Ku_WT_ described above.

### A Ku–LIG–DNA complex forms independently of the Ku C-terminus

**Figure 12.**
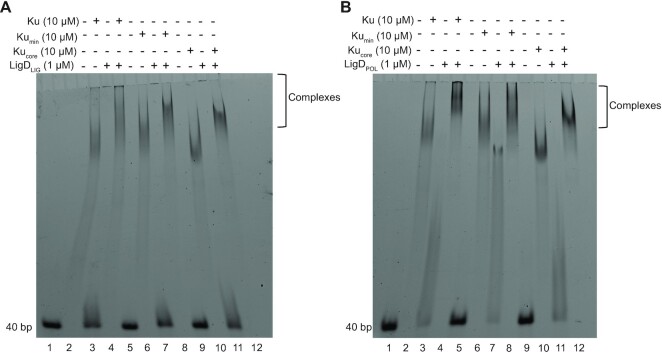
LigD LIG and LigD POL form ternary complexes with DNA and Ku_WT_, Ku_min_ or Ku_core_. 40 bp dsDNA was incubated with 10 μM of Ku_WT_, Ku_min_ and Ku_core_, and/or 1 μM LigD POL or LIG, as indicated above and analysed by EMSA. Ku-LIG/POL-DNA complexes are identified based on band migration compared to the individual proteins alone. This image is a representative gel from three independent experiments, visualized by a 6% non-denaturing PAGE.

Given that Ku can bind LIG and POL separately, we wanted to investigate if the individual domains are necessary for a Ku–LigD–DNA ternary complex formation and if the Ku C-terminus affects this formation. Using the same EMSA as described earlier for a ternary Ku–LigD–DNA complex, we saw that Ku_WT_, Ku_min_ and Ku_core_ form super-shifted complexes with both LIG and POL, similar to LigD (Figure 12). Therefore, even though LIG and POL have a weaker affinity for Ku_min_ and Ku_core_ compared to Ku_WT_ by BLI, the presence of DNA may stabilize the interaction, and allow the ternary complex to form.

## DISCUSSION

**Figure 13.**
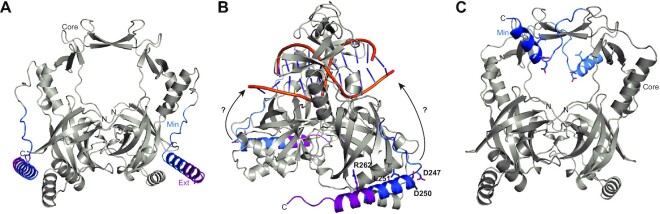
ColabFold predicted structures of homodimeric Ku (22,23,36). (**A**) Ku_WT_ homodimer. Core, grey; minimal C-terminal region, blue; extended C-terminal region, purple. (**B**) Ku_WT_ homodimer with a dsDNA substrate manually modelled in the protein to illustrate how DNA may bind through the Ku core domain. Conserved residues linked to DNA binding activity are shown as sticks. Arrows suggest potential movement of the Ku C-terminus. Colours are as in (A). dsDNA substrate is from PDB 4FZY. (**C**) Ku_min_ homodimer, coloured as in (A), with conserved residues linked to DNA binding activity shown as sticks.

The impact of the C-terminal region of Ku in *M. tuberculosis* on LigD ligation is unclear, given its significantly shortened length in the extended region, compared to similarly studied Ku homologues in other bacterial species like *B. subtilis* (13). In this study, we take a quantitative approach to unravel the mechanism of how changes in the *M. tuberculosis* Ku C-terminus affect Ku–DNA binding, Ku-stimulated ligation by LigD, and the affinity of Ku in a direct interaction with LigD, to gain insight into how these activities may come together during NHEJ. First, we find that *M. tuberculosis* Ku can bind as little as 15bp, whether it is Ku_WT_ or the core domain alone, although more stable interactions are formed with longer substrates, with Ku_min_ and Ku_core_ having a higher affinity than Ku_WT_ for longer DNA substrates of 30–40 bp (Figure 2). This same trend has been observed previously with *M. tuberculosis* Ku and *B. subtilis* Ku, the latter of which also has a 15bp footprint for Ku_WT_ and ∼11 bp footprint for Ku_core_ (4,12). Our results are also consistent with data showing that the core domain of *B. subtilis* Ku can bind DNA, similar to the eukaryotic Ku homolog, Ku70/80 (12,21). Based on an *in silico* model of *M. tuberculosis* Ku predicted by ColabFold (Figure 13A and B), it is clear that DNA likely threads through the central pore, identical to how DNA threads through Ku70/80 (21). However, in *B. subtilis*, the added Ku C-terminus improves DNA binding (12), while results shown here for *M. tuberculosis* are the opposite. The presence of the C-terminus of *M. tuberculosis* Ku reduces, although does not fully inhibit DNA binding (Figure 2), yet the presence of the C-terminus is needed for efficient bridging activity, allowing Ku to form a synapse between two DNA molecules (Figure 4). How do we reconcile these apparently contradictory results? In NHEJ, a reduced, or regulated DNA binding affinity is necessary. Ku from *B. subtilis* translocates along DNA, with the same function also proposed for *M. tuberculosis* Ku (4,12,13). Therefore, a strong Ku–DNA interaction would be problematic, preventing movement of Ku along the DNA and blocking recruitment and binding of LigD at a DSB. The C-terminus of Ku, which reduces the overall affinity of Ku for DNA (Figure 2), is therefore critical for regulating Ku–DNA binding. We identified specific amino acids that could be involved in this regulatory role, such as Ku L251, where a mutation to alanine reduced the Ku–DNA binding affinity, indicating L251 promotes DNA binding (Figure 3). Leucine, however, is not a likely candidate for a direct DNA interaction due to its hydrophobic nature, suggesting that L251 may indirectly affect DNA binding by stabilizing an optimal structural configuration of Ku that increases affinity for DNA. These attractive forces would be balanced by D247 and D250, which when mutated to alanine, had increased DNA binding, suggesting that D247 and D250 limit DNA binding affinity in Ku_WT_, likely through electrostatic repulsion of the negatively charged DNA phosphate backbone. Interestingly, the positively charged residue R262 would theoretically attract DNA, however our results show R262 limits DNA binding. While not entirely clear, it is possible that R262 plays an indirect role like L251, either by destabilizing the DNA binding configuration of Ku, or that the mutation to alanine created a more optimal Ku–DNA binding configuration than Ku_WT_. Further evidence, such as a DNA-bound Ku structure, is needed to determine if this hypothesis is correct. Meanwhile, these DNA binding regulatory residues are in a predicted alpha helix in the Ku C-terminus and appear tightly packed against the predicted Ku structure (Figure 13B). This predicted alpha helix is connected by a long loop to the Ku core domain, suggesting that there is flexibility, and that this helix could be repositioned to help in binding and bridging DNA, particularly if the DNA was bound through the central pore, as modelled in Figure 13B.

Single-molecule studies of Ku and LigD from *B. subtilis* suggested there were additional interactions between DNA-bound Ku, independent of the C-terminal arms, although studies of the *B. subtilis* Ku core domain did not form a supershifted complex in an EMSA with LigD and DNA. These findings are contrary to what we observed for *M. tuberculosis* Ku_core_, LigD and DNA, where we observe a supershifted complex of the Ku core domain with LigD on DNA (Figure 8) (12). Additionally, we found that *M. tuberculosis* Ku and LigD directly interact not only through both the extended and minimal C-terminus, but through the core domain in the absence of DNA, by BLI (Figure 7A and B). Interestingly, though, the Ku_core_ stimulated ligation of blunt- and sticky-ended DNA substrates, while inhibiting LigD nick-sealing activity (Figures 5A–D and 6A, B). The core of *P.aeruginosa* Ku is also capable of stimulating ligation of a linearised plasmid with complementary overhangs (20), but why does this effect of the Ku_core_ on plasmid ligation not extend to nicks, and even become inhibitory? It is likely a combination of the high affinity of the Ku_core_ for DNA and the shorter length of the nicked DNA substrate, leading to Ku_core_ blocking access to the nicked DNA. These results also indirectly show that Ku translocates along DNA. With the linearised plasmid substrates used for sticky and blunt-ended ligation, an energetically favourable interaction between Ku and DNA is maintained, even if Ku moves away from the break site, as Ku remains in contact with the maximal number of nucleotides. With the nicked DNA substrate, only 36bp long, it is likely that the any large translocations of Ku_core_ are energetically unfavourable, as the Ku_core_ - DNA interaction weakens as the DNA substrate length decreases (Figure 2E and F, Supplementary Table S5). Therefore, Ku_core_ likely remains relatively immobile on the nicked DNA, preventing LigD from accessing the damage, and suggests that Ku-stimulated ligation is a balance between Ku–DNA binding affinity and Ku–LigD interaction affinity.

We noticed an interesting trend in some of our results, where Ku_min_ had a poorer outcome than Ku_core_. For example, Ku_min_ was less effective at DNA bridging, compared to Ku_core_ (Figure 4), almost as if the shorter C-terminus was inhibiting activity, even though Ku_min_ has a higher DNA binding affinity compared to Ku_WT_ (Figure 2). The affinity of Ku_min_ for directly binding LigD was also less than both Ku_WT_ and Ku_core_ (Figure 7). However, the weakened affinity had no effect on the formation of a DNA–Ku_min_–LigD complex (Figure 8). Based on the ColabFold predicted structure of Ku_WT_, the minimal C-terminus encompasses the flexible loop and about half of the alpha helix that makes up the remaining C-terminal structure. This shortened alpha-helix structure is predicted by ColabFold to remain when the extended C-terminus is removed (Figure 13C) (23,36). This shorter alpha helix attached to a flexible loop may be able to bind and block areas that the longer alpha helix with the extended Ku C-terminus does not, whether that be a region of LigD important for a stable Ku–LigD interaction, or on Ku that is needed for stable synapsis of two DNA molecules (Figure 13C). Future structural studies will be required to determine if this is the case.

While much of our work focused on wildtype LigD and Ku C-terminal truncations, we also studied Ku-stimulated ligation and interactions with the individual ligase and polymerase domains of LigD. The ligase domain LIG was incapable of ligating the longer dsDNA substrates sufficiently for accurate results when Ku was added (Figure 9). However, with LIG and nick-sealing activity on the shorter DNA substrate, we found that removal of the extended Ku C-terminus, leaving behind only the minimal C-terminus, produces the highest LIG nick-sealing rate compared to LIG alone, while Ku_WT_ has no effect (Figure 10). How does this align with our results that Ku_WT_ stimulates higher rates of nick-sealing ligation with LigD than Ku_min_, Ku_core_ or LigD alone? One possible mechanism is that in the absence of the other LigD domains, the disordered, flexible extended Ku C-terminus may physically impede the LIG active site, since Ku_WT_ still tightly binds directly to LIG (Figure 11). However, when the entire LigD protein is present, (i.e. LIG, POL and phosphoesterase domains), the Ku extended C-terminus could act as an allosteric effector, binding elsewhere on LigD to stimulate nick-sealing. The polymerase domain POL is an attractive allosteric binding site, given previous research suggesting that the Ku–LigD interaction occurs primarily through the POL domain (17), and from the work presented here, where Ku_WT_ binds tightly to POL, compared to when the extended or entire C-terminus is removed, resulting in decreased affinities for POL (Figure 11).

While the extended C-terminus may be an allosteric stimulator of ligation, we also note that a direct interaction between Ku and LIG may still occur to stimulate ligation, but rather through the core domain of Ku. Ku_core_ directly binds to LIG, albeit not as strongly as Ku_WT,_ but importantly, Ku_core_ binds LigD with the same affinity as Ku_WT_ (Figures 7A, B, 11A, B). Additionally, Ku_core_ stimulates the rate of dsDNA ligation higher than LigD alone (Figure 5), while Ku_core_ also stimulates nick-sealing ligation to rates faster than LIG alone or with Ku_WT_ (Figure 10), suggesting that direct interaction between Ku_core_ and LigD may also stimulate ligation, in addition to the DNA-binding and bridging contributions of the Ku_core_ (Figures 2 and 4).

As mentioned earlier, the Ku-stimulated LigD ligation mechanism is likely a balance between: 1) a direct Ku–LigD interaction, and 2) the Ku–DNA binding affinity and DNA length, which in turn could affect Ku translocation. As an example, consider the ligation of longer, dsDNA, where Ku_WT_ stimulates the fastest rate of ligation (Figure 5). This ligation corresponds with a strong protein-protein interaction affinity as measured by BLI, (Figure 7), yet Ku_WT_ has the weakest affinity for DNA compared to the other Ku truncations (Figure 2). This balance is also seen in our Ku point mutants. We inadvertently created mutant Ku proteins that directly bound LigD with higher affinity than Ku_WT_ (Ku L251A, R262A, Figure 7), yet Ku L251A, part of the minimal C-terminus, had a weaker affinity for DNA and Ku R262A, part of the extended C-terminus, had a stronger affinity for DNA compared to Ku_WT_ (Figure 3). In terms of ligation, neither had any stimulatory effect on dsDNA ligation, yet both mutants were able to moderately stimulate LigD nick-sealing rates higher than Ku_WT_ (Figures 5 and 6).

A second example of this balance can be seen with nick-sealing rates of the 36bp DNA substrates. We found that the extended Ku C-terminus (Ku_WT_) is needed for the highest rate of nick-sealing, as removal of this region reduced rates to those of LigD alone (Figure 6). Taking into consideration this balance, we know that Ku_WT_ has the weakest DNA binding affinity for 30–40 bp dsDNA, compared to Ku_min_ and Ku_core_ (Figure 2), therefore it would more readily translocate along the nicked DNA making room for LigD to repair the nick, but Ku_WT_ has the strongest affinity for a direct interaction with LigD, thus stimulating ligation. Shorter C-terminal truncations of Ku have tighter binding to the 30–40 bp dsDNA, and thus would hinder translocation, and in turn, nick-sealing activity, as evidenced for lower rates of nick-sealing by Ku_min_ and Ku_core_.

**Figure 14.**
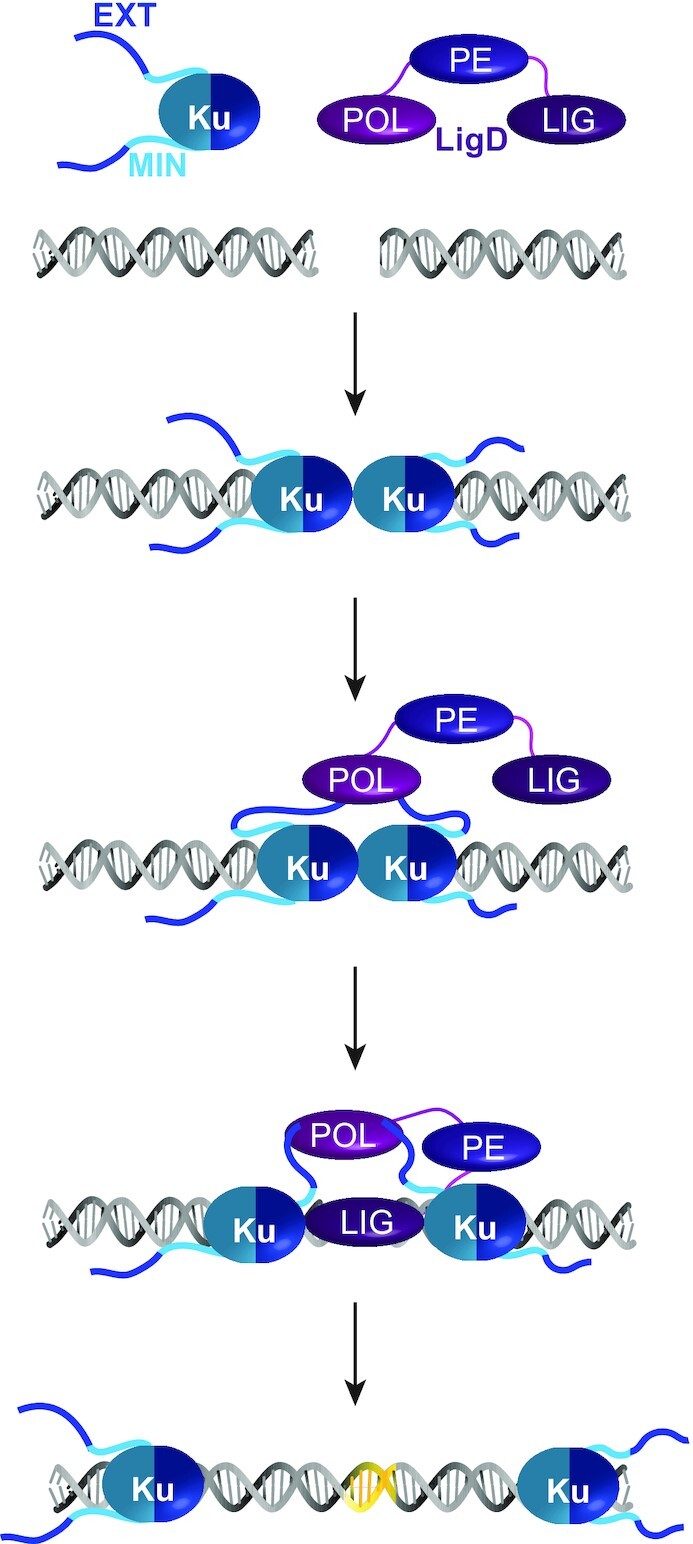
Working model of how the Ku C-terminus contributes to NHEJ. When a DNA DSB is formed, the Ku core domain and minimal C-termini bind to DNA and bridge the DNA ends. The Ku extended C-terminus helps recruit LigD, likely through interactions with the POL domain. To ligate the DNA DSB, Ku translocates down the DNA to make room for LigD, specifically LIG at the break site. LIG directly interacts with the Ku core domain, while the Ku extended C-termini maintain contact with POL. Combined, these interactions stimulate ligation of the DSB, leaving behind a repaired DNA DSB. MIN, Ku minimal C-terminus in blue; EXT, Ku extended C-terminus in purple; LIG, ligase domain of LigD; PE, phosphoesterase domain of LigD; POL, polymerase domain of LigD.

While many questions remain, what we can conclude from our data is that both the minimal conserved and extended variable regions of the *M. tuberculosis* Ku C-terminus play multiple roles, characteristic of regions containing intrinsic disorder (37). The Ku extended C-terminus is partly responsible for the direct interaction between Ku_WT_ and LigD, as suggested by BLI (Figure 7), and is needed for maximal DNA bridging (Figure 4), leading to stimulated ligation rates (Figure 5). However, the Ku extended C-terminus also limits DNA binding (Figure 2), presumably to aid in translocation along DNA as discussed above. The minimal, conserved C-terminus of Ku increases DNA binding affinity, with amino acids (L251, S258) that promote favourable Ku–DNA interactions, and although Ku_min_ has a lower affinity for LigD (Figure 7), Ku_min_ is still sufficient to stimulate high rates of sticky and blunt-ended ligation (Figure 5). These functions align with the multi-purpose functionality of Ku in NHEJ. We propose a model where Ku must initially bind the DSB, but with a low enough affinity to translocate for LigD binding. At the DSB, Ku can bridge the DNA ends through the core and minimal C-terminus (Figure 14), with possibly two Ku homodimers bridging the ends, similar to what was observed for *B. subtilis* Ku, where a minimum of two Ku homodimers were required for stable bridging (12). Meanwhile, the Ku extended C-terminus helps recruit LigD to the break, through a direct interaction. Where the extended Ku C-terminus binds LigD may be flexible and dependent on the processing required by the DNA ends before ligation. We suggest here the combination of an allosteric and direct-binding mechanism for stimulating ligation based on our studies with the individual LIG and POL domains of LigD, where the core domain of Ku binds directly to LIG, while the extended C-terminus of Ku binds to POL to stimulate ligation. *P. aeruginosa* Ku can also stimulate the polymerase activity of LigD (20), therefore it is possible that a similar allosteric mechanism may occur when nucleotides need to be added to the DNA ends and the Ku extended C-terminus binds the ligase domain to stimulate polymerisation. Our data showing the direct interaction of Ku with LigD POL supports such a theory (Figure 11), as does evidence from others based on both *M. tuberculosis* Ku and *B. subtilis* Ku interacting with LigD POL (12,13,17). Overall, these results would suggest that the Ku–LigD interaction is fluid and dependent on whether the DNA ends require nucleotide addition or ligation. Future structural studies of Ku–LigD in complex with a variety of DNA substrates will be of great interest in further understanding this mechanism.

In summary, we have shown that the Ku C-terminus of *M. tuberculosis* is crucial for stimulating ligation by LigD through direct protein-protein interactions and Ku–DNA binding. Given that the Ku C-terminus is also unique to bacteria, targeting the Ku C-terminus may be a useful strategy in future antimicrobial drug development, particularly against tuberculosis. Future studies *in vivo* are needed to determine if the results shown here play a significant role in DNA repair biologically, but given the DNA damage potential of antibiotics, targeting DNA repair pathways may prove a useful method to increase the efficacy of current antimicrobials.

## DATA AVAILABILITY

The raw images and datasets analysed in the current study are available from the corresponding author upon reasonable request.

## Supplementary Material

gkac906_Supplemental_File

## Notes

Present address: Jose Pablo Rascon Perez, Department of Biology, McGill University, Montreal, Quebec H3A 0G4, Canada.

Present address: Cody Caba, Department of Chemistry and Biochemistry, University of Windsor, Windsor, Ontario N9B 3P4, Canada.

## References

[1] Wyman C. , Kanaar R. DNA double-strand break repair: all's well that ends well. Annu. Rev. Genet. 2006; 40:363–383. 16895466 10.1146/annurev.genet.40.110405.090451

[2] Aravind L. , Koonin E.V. Prokaryotic homologs of the eukaryotic DNA-end-binding protein Ku, novel domains in the Ku protein and prediction of a prokaryotic double-strand break repair system. Genome Res. 2001; 11:1365–1374. 11483577 10.1101/gr.181001 PMC311082

[3] Doherty A.J. , Jackson S.P., Weller G.R. Identification of bacterial homologues of the Ku DNA repair proteins. FEBS Lett. 2001; 500:186–188. 11445083 10.1016/s0014-5793(01)02589-3

[4] Weller G.R. , Kysela B., Roy R., Tonkin L.M., Scanlan E., Della M., Devine S.K., Day J.P., Wilkinson A., di Fagagna F. et al. Identification of a DNA nonhomologous end-joining complex in bacteria. Science. 2002; 297:1686–1689. 12215643 10.1126/science.1074584

[5] Della M. , Palmbos P.L., Tseng H.-M., Tonkin L.M., Daley J.M., Topper L.M., Pitcher R.S., Tomkinson A.E., Wilson T.E., Doherty A.J. Mycobacterial Ku and ligase proteins constitute a two-component NHEJ repair machine. Science. 2004; 306:683–685. 15499016 10.1126/science.1099824

[6] Gong C. , Bongiorno P., Martins A., Stephanou N.C., Zhu H., Shuman S., Glickman M.S. Mechanism of nonhomologous end-joining in mycobacteria: a low-fidelity repair system driven by Ku, ligase D and ligase C. Nat. Struct. Mol. Biol. 2005; 12:304–312. 15778718 10.1038/nsmb915

[7] Shuman S. , Glickman M.S. Bacterial DNA repair by non-homologous end joining. Nat. Rev. Microbiol. 2007; 5:852–861. 17938628 10.1038/nrmicro1768

[8] Stephanou N.C. , Gao F., Bongiorno P., Ehrt S., Schnappinger D., Shuman S., Glickman M.S. Mycobacterial nonhomologous end joining mediates mutagenic repair of chromosomal double-strand DNA breaks. J. Bacteriol. 2007; 189:5237–5246. 17496093 10.1128/JB.00332-07 PMC1951864

[9] de Vega M. , Vega M.de The minimal bacillus subtilis nonhomologous end joining repair machinery. PLoS One. 2013; 8:e64232. 23691176 10.1371/journal.pone.0064232 PMC3656841

[10] Sinha K.M. , Stephanou N.C., Gao F., Glickman M.S., Shuman S. Mycobacterial UvrD1 is a Ku-dependent DNA helicase that plays a role in multiple DNA repair events, including double-strand break repair. J. Biol. Chem. 2007; 282:15114–15125. 17376770 10.1074/jbc.M701167200

[11] Li Z. , Wen J., Lin Y., Wang S., Xue P., Zhang Z., Zhou Y., Wang X., Sui L., Bi L-J. et al. A Sir2-like protein participates in mycobacterial NHEJ. PLoS One. 2011; 6:e20045. 21637345 10.1371/journal.pone.0020045 PMC3102665

[12] Öz R. , Wang J.L., Guerois R., Goyal G., KK S., Ropars V., Sharma R., Koca F., Charbonnier J.-B., Modesti M. et al. Dynamics of Ku and bacterial non-homologous end-joining characterized using single DNA molecule analysis. Nucleic Acids Res. 2021; 49:2629–2641. 33590005 10.1093/nar/gkab083 PMC7969030

[13] McGovern S. , Baconnais S., Roblin P., Nicolas P., Drevet P., Simonson H.H., Pietrement O., Charbonnier J.-B.B., Cam E.Le, Noirot P. et al. C-terminal region of bacterial Ku controls DNA bridging, DNA threading and recruitment of DNA ligase D for double strand breaks repair. Nucleic Acids Res. 2016; 44:4785. 26961308 10.1093/nar/gkw149 PMC4889933

[14] De Ory A. , Zafra O., de Vega M. Efficient processing of abasic sites by bacterial nonhomologous end-joining Ku proteins. Nucleic Acids Res. 2014; 42:13082–13095. 25355514 10.1093/nar/gku1029 PMC4245934

[15] Roberts S.A. , Strande N., Burkhalter M.D., Strom C., Havener J.M., Hasty P., Ramsden D.A. Ku is a 5′-dRP/AP lyase that excises nucleotide damage near broken ends. Nature. 2010; 464:1214–1217. 20383123 10.1038/nature08926 PMC2859099

[16] Weller G.R. , Doherty A.J. A family of DNA repair ligases in bacteria. FEBS Lett. 2001; 505:340–342. 11566200 10.1016/s0014-5793(01)02831-9

[17] Pitcher R.S. , Tonkin L.M., Green A.J., Doherty A.J. Domain structure of a NHEJ DNA repair ligase from mycobacterium tuberculosis. J. Mol. Biol. 2005; 351:531–544. 16023671 10.1016/j.jmb.2005.06.038

[18] Zhu H. , Shuman S. A primer-dependent polymerase function of pseudomonas aeruginosa ATP-dependent DNA ligase (LigD). J. Biol. Chem. 2005; 280:418–427. 15520014 10.1074/jbc.M410110200

[19] Zhu H. , Shuman S. Novel 3′-ribonuclease and 3′-phosphatase activities of the bacterial non-homologous end-joining protein, DNA ligase D. J. Biol. Chem. 2005; 280:25973–25981. 15897197 10.1074/jbc.M504002200

[20] Zhu H. , Shuman S. Gap filling activities of pseudomonas DNA ligase D (LigD) polymerase and functional interactions of LigD with the DNA end-binding Ku protein. J. Biol. Chem. 2010; 285:4815–4825. 20018881 10.1074/jbc.M109.073874 PMC2836087

[21] Walker J.R. , Corpina R.A., Goldberg J. Structure of the Ku heterodimer bound to DNA and its implications for double-strand break repair. Nature. 2001; 412:607–614. 11493912 10.1038/35088000

[22] Amare B. , Mo A., Khan N., Sowa D.J., Warner M.M., Tetenych A., Andres S.N. LigD: a structural guide to the multi-tool of bacterial non-homologous end joining. Front. Mol. Biosci. 2021; 8:1161. 10.3389/fmolb.2021.787709 PMC8656161 34901162

[23] Jumper J. , Evans R., Pritzel A., Green T., Figurnov M., Ronneberger O., Tunyasuvunakool K., Bates R., Žídek A., Potapenko A. et al. Highly accurate protein structure prediction with AlphaFold. Nature. 2021; 596:583–589. 34265844 10.1038/s41586-021-03819-2 PMC8371605

[24] Kushwaha A.K. , Grove A. C-terminal low-complexity sequence repeats of *Mycobacterium**smegmatis* Ku modulate DNA binding. Biosci. Rep. 2013; 33:e00016. 23167261 10.1042/BSR20120105 PMC3553676

[25] Stols L. , Gu M., Dieckman L., Raffen R., Collart F.R., Donnelly M.I. A new vector for high-throughput, ligation-independent cloning encoding a tobacco etch virus protease cleavage site. Protein Expr. Purif. 2002; 25:8–15. 12071693 10.1006/prep.2001.1603

[26] Eschenfeldt W.H. , Stols L., Sanville Millard C., Joachimiak A., Donnelly M.I. A family of LIC vectors for high-throughput cloning and purification of proteins. Methods Mol. Biol. 2009; 498:105–115. 18988021 10.1007/978-1-59745-196-3_7 PMC2771622

[27] Zheng L. , Baumann U., Reymond J.-L.L. An efficient one-step site-directed and site-saturation mutagenesis protocol. Nucleic Acids Res. 2004; 32:e115. 15304544 10.1093/nar/gnh110 PMC514394

[28] Schneider C.A. , Rasband W.S., Eliceiri K.W. NIH Image to Imagej: 25 years of image analysis. Nat. Methods. 2012; 9:671–675. 22930834 10.1038/nmeth.2089 PMC5554542

[29] Andres S.N. , Appel C.D., Westmoreland J.W., Williams J.S., Nguyen Y., Robertson P.D., Resnick M.A., Williams R.S. Tetrameric Ctp1 coordinates DNA binding and DNA bridging in DNA double-strand-break repair. Nat. Struct. Mol. Biol. 2015; 22:158–166. 25580577 10.1038/nsmb.2945 PMC4318798

[30] Zhu H. , Shuman S. Bacterial nonhomologous end joining ligases preferentially seal breaks with a 3′-OH monoribonucleotide. J. Biol. Chem. 2008; 283:8331–8339. 18203718 10.1074/jbc.M705476200 PMC2276377

[31] Gong C. , Martins A., Bongiorno P., Glickman M., Shuman S. Biochemical and genetic analysis of the four DNA ligases of mycobacteria. J. Biol. Chem. 2004; 279:20594–20606. 14985346 10.1074/jbc.M401841200

[32] Zhu H. , Shuman S. Characterization of agrobacterium tumefaciens DNA ligases C and D. Nucleic Acids Res. 2007; 35:3631–3645. 17488851 10.1093/nar/gkm145 PMC1920237

[33] Sélo I. , Négroni L., Créminon C., Grassi J., Wal J.M. Preferential labeling of alpha-amino groups in peptides by biotin: application to the detection of specific anti-peptide antibodies by enzyme immunoassays. J. Immunol. Methods. 1996; 199:127–138. 8982354 10.1016/s0022-1759(96)00173-1

[34] Gasteiger E. , Hoogland C., Gattiker A., Duvaud S., Wilkins M.R., Appel R.D., Bairoch A. Walker J.M. Protein analysis tools on the expasy server. The Proteomics Protocols Handbook. 2005; Humana Press 988.

[35] Oates M.E. , Romero P., Ishida T., Ghalwash M., Mizianty M.J., Xue B., Dosztanyi Z., Uversky V.N., Obradovic Z., Kurgan L. et al. D(2)P(2): database of disordered protein predictions. Nucleic Acids Res. 2013; 41:D508–D516. 23203878 10.1093/nar/gks1226 PMC3531159

[36] Mirdita M. , Schutze K., Moriwaki Y., Heo L., Ovchinnikov S., Steinegger M. ColabFold: making protein folding accessible to all. Nat. Methods. 2022; 19:679–682. 35637307 10.1038/s41592-022-01488-1 PMC9184281

[37] Babu M.M. , Kriwacki R.W., Pappu R.V Structural biology. Versatility from protein disorder. Science. 2012; 337:1460–1461. 22997313 10.1126/science.1228775

[38] Sievers F. , Wilm A., Dineen D., Gibson T.J., Karplus K., Li W., Lopez R., McWilliam H., Remmert M., Söding J. et al. Fast, scalable generation of high-quality protein multiple sequence alignments using clustal omega. Mol. Syst. Biol. 2011; 7:539. 21988835 10.1038/msb.2011.75 PMC3261699

