# Supplementary material for: The Mycobacterium tuberculosis Ku C-terminus is a multi-purpose arm for binding DNA and LigD and stimulating ligation
Source: Nucleic Acids Res. 2022 Oct 17;50(19):11040–57. doi: 10.1093/nar/gkac906 (PMC9638933; doi:10.1093/nar/gkac906)
Supplement: gkac906_Supplemental_File [file gkac906_supplemental_file.pdf]

**Supplementary Material**

**Supplementary Table S1:** Cloning Primers for Protein Expression Plasmids

| Protein expression construct | Oligonucleotides | Plasmid Backbone | Cloning Method |
|---|---|---|---|
| Ku, wildtype | 5’ TACTTCCAATCCAATGAAATGCGTG 3’<br>5’ TTATCCACTTCCAATGTTATTATGGCG 3’ | pMCSG7(1) | Ligation independent cloning (LIC)(1) |
| $Ku_{min}$ | 5’ TACTTCCAATCCAATGAAATGCGTG 3’<br>5’ TTATCCACTTCCAATGTTATCAGACACTCG CTTCTAACTTTG 3’ | pMCSG7(1) | LIC(1) |
| $Ku_{core}$ | 5’ TACTTCCAATCCAATGAAATGCGTG 3’<br>5’ TTATCCACTTCCAATGTTATCACTGATCCT CGGCTGTGAAG 3’ | pMCSG7(1) | LIC(1) |
| Ku D247A | 5’ GACGAGCCGGAGGCTGTGTCGGATTTAC 3’<br>5’ CACATCCTCCGGCTCGTCAAG 3’ | pMCSG7(1) | Site-directed mutagenesis (SDM)(2) |
| Ku D250A | 5’ GGAGGATGTGTCGGCTTTACTTGCAAAGTT AGAAGC 3’<br>5’ CACATCCTCCGGCTCGTCAAG 3’ | pMCSG7(1) | SDM(2) |
| Ku L251A | 5’ GGAGGATGTGTCGGATGCACTTGCAAAGTT AGAAGCG 3’<br>5’ CACATCCTCCGGCTCGTCAAG 3’ | pMCSG7(1) | SDM(2) |
| Ku L255A | 5’ GATTTACTTGCAAAGGCAGAAGCGAGTGTC AAAGCCC 3’<br>5’ CAAGTAAATCCGACACATCCTCCGGC 3’ | pMCSG7(1) | SDM(2) |
| Ku S258A | 5’ GATTTACTTGCAAAGTTAGAAGCGGCCGTC AAAGCCCGTTCC 3’<br>5’ CAAGTAAATCCGACACATCCTCCGGC 3’ | pMCSG7(1) | SDM(2) |
| Ku R262A | 5’ GCGAGTGTCAAAGCCGCCTCCAAAGCTAAC TCTAATGTTCC 3’<br>5’ GACACTCGCTTCTAACTTTGCAAG 3’ | pMCSG7(1) | SDM(2) |
| LigD | 5’ TACTTCCAATCCAATGAAATGGGC 3’<br>5’ TTATCCACTTCCAATGTTATTACTCGC 3’ | pMCSG7(1) | LIC(1) |
| POL | 5’ GAACGCTTGGATTAATAAGCCCCCGTGGCT GAC 3’<br>5’ CCAAGCGTTCTAACAAATCCCCGTC 3’ | pMCSG7(1) | SDM(2) |
| LIG | 5’ GAATTCGATAATCTGGCGCCCATG 3’<br>5’ AGATTATCGAATTCCATTTCATTGGATTGG AAGTACAGG 3’ | pMCSG7(1) | SDM(2) |

**Supplementary Table S2:** Oligonucleotide Sequences for DNA Binding Substrates

| Figure Panel | Substrate | Oligonucleotides |
|---|---|---|
| S11 A-B | 10bp dsDNA | 5' 6-FAM CAGGAAGGTG 3'<br>5' CACCTTCCTG 3' |
| 2 A-F, S11 A-B | 15bp dsDNA | 5' 6-FAM CAGGAAGGGAATGTT 3'<br>5' AACATTCCCTTCCTG 3' |
| 2 A-F, S11 A-B | 20bp dsDNA | 5' 6-FAM CAGGAAGGGAATGTTGTCTG 3'<br>5' CAGACAACATTCCCTTCCTG 3' |
| 2 A-F, S11 A-B | 30bp dsDNA | 5' 6-FAM CAGGAGGTGTAGGGAAGGGAATGTTGTCTG 3'<br>5' CAGACAACATTCCCTTCCCTACACCTCCTG 3' |
| 2 A-F, 3 A-C, S11 A-B | 40bp dsDNA | 5' 6-FAM CAGGGTAAGTGTGGAGGTGTAGGGAAGGGAATGTTGTCTG 3'<br>5' CAGACAACATTCCCTTCCCTACACCTCCACACTTACCCTG 3' |
| 6 A-D, 10 A-B, S9, S10, S12 | 36bp nicked DNA | 5' (PO4) TCATCGAGGGATTACATC 6-FAM 3'<br>5' CAACTGCAGTTCTAGACC 3'<br>5' GATGTAATCCCTCGATGAGGTCTAGAACTGCAGTTG 3' |

**Supplementary Table S3:** Biotinylated Sites of $Ku_{WT}$, $Ku_{min}$, and $Ku_{core}$ for Bio-layer Interferometry Analysis

| Lysine | $Ku_{WT}$ (1-273) | $Ku_{min}$ (1-259) | $Ku_{core}$ (1-238) |
|---|---|---|---|
| K19 | B | B | B |
| K37 | B | B | B |
| K45 | B | B | B |
| K117 | B | B | B |
| K120 | B | B | B |
| K127 | B | B | B |
| K145 | N | N | N |
| K154 | N | B | B |
| K158 | N | N | B |
| K184 | B | B | B |
| K188 | B | B | B |
| K193 | B | B | B |
| K226 | B | B | B |
| K254 | B | B | X |
| K260 | B | X | X |
| K264 | B | X | X |

B, biotinylated; N, not biotinylated; X, Lys not in this construct

**Supplementary Table S4:** Molecular Weight and Oligomeric State from SEC-MALS

| Protein | Theoretical MW (kDa) | MALS MW (kDa) | Oligomeric State |
|---|---|---|---|
| Ku | 33.7 | 70.6 | Dimer |
| $Ku_{min}$ | 32.3 | 75.2 | Dimer |
| $Ku_{core}$ | 30.0 | 68.8 | Dimer |
| Ku D247A | 33.7 | 65.8 | Dimer |
| Ku D250A | 33.7 | 67.1 | Dimer |
| Ku L251A | 33.7 | 63.0 | Dimer |
| Ku L255A | 33.7 | 85.1 | Undetermined |
| Ku S258A | 33.7 | 68.1 | Dimer |
| Ku R262A | 33.6 | 58.4 | Dimer |
| LigD | 86.4 | 94.2 | Monomer |
| LigD POL | 34.0 | 32.8 | Monomer |
| LigD LIG | 36.6 | 33.9 | Monomer |

**Supplementary Table S5:** Ku-DNA Binding Affinity Apparent $K_D$ Values

| | $K_D$ ± SEM (μM) | | | |
|---|---|---|---|---|
| **Protein** | **15bp** | **20bp** | **30bp** | **40bp** |
| Ku | 1.63 (±0.30) | 4.76 (±1.24) | 1.18 (±0.09) | 3.15 (±0.74) |
| $Ku_{min}$ | 0.97 (±0.15) | 0.90 (±0.08) | 0.30 (±0.14) | 0.46 (±0.08) |
| $Ku_{core}$ | 0.41 (±0.04) | 0.20 (±0.01) | 0.19 (±0.01) | 0.07 (±0.01) |
| D247A | n/a | n/a | n/a | 0.16 (±0.01) |
| D250A | n/a | n/a | n/a | 0.14 (±0.01) |
| L251A | n/a | n/a | n/a | 1.30 (±0.19) |
| S258A | n/a | n/a | n/a | 1.34 (±0.29) |
| R262A | n/a | n/a | n/a | 0.14 (±0.01) |

**Supplementary Table S6:** Ligation Rates for dsDNA Ligation, Sticky Ends (*KpnI*)

| Protein 1 | Protein 2 | Rate±SD (pmol/min) |
|---|---|---|
| LigD | - | 29.6 (±0.6) |
| LigD | $Ku_{WT}$ | 58.4 (±6.3) |
| LigD | $Ku_{min}$ | 61.9 (±2.3) |
| LigD | $Ku_{core}$ | 41.5 (±6.1) |
| LigD | Ku D247A | 16.8 (±0.7) |
| LigD | Ku D250A | 14.6 (±1.7) |
| LigD | Ku L251A | 19.3 (±1.1) |
| LigD | Ku S258A | 15.6 (±5.1) |
| LigD | Ku R262A | 23.9 (±2.0) |

**Supplementary Table S7:** Ligation Rates for dsDNA Ligation, Blunt Ends (*SmaI*)

| Protein 1 | Protein 2 | Rate±SD (pmol/min) |
|---|---|---|
| LigD | - | 29.6 (±2.2) |
| LigD | $Ku_{WT}$ | 56.4 (±2.3) |
| LigD | $Ku_{min}$ | 59.6 (±4.4) |
| LigD | $Ku_{core}$ | 48.2 (±2.1) |
| LigD | Ku D247A | 16.1 (±1.6) |
| LigD | Ku D250A | 15.1 (±0.9) |
| LigD | Ku L251A | 15.3 (±5.8) |
| LigD | Ku S258A | 14.7 (±3.1) |
| LigD | Ku R262A | 20.9 (±1.2) |

**Supplementary Table S8:** Nick-Sealing Ligation Rates

| Protein 1 | Protein 2 | Rate ± SD (pM/min) |
|---|---|---|
| LigD | - | 27.8 (±4.1) |
| LigD | Ku | 37.3 (±1.3) |
| LigD | $Ku_{min}$ | 26.8 (±1.3) |
| LigD | $Ku_{core}$ | 4.5 (±2.0) |
| LigD | Ku D247A | 23.6 (±8.8) |
| LigD | Ku D250A | 28.5 (±6.8) |
| LigD | Ku L251A | 41.3 (±2.2) |
| LigD | Ku S258A | 28.8 (±8.6) |
| LigD | Ku R262A | 62.1 (±6.2) |
| LIG | - | 7.9 (±0.3) |
| LIG | Ku | 8.2 (±1.8) |
| LIG | $Ku_{min}$ | 28.7 (±7.7) |
| LIG | $Ku_{core}$ | 19.7 (±2.9) |

**Supplementary Table S9:** Protein-Protein Interaction Affinity $K_D$ Values

| Ligand | Analyte | $K_D$ ± SD (nM) |
|---|---|---|
| Ku | LigD | 196 (±5.0) |
| $Ku_{min}$ | LigD | 288 (±1.4) |
| $Ku_{core}$ | LigD | 200 (±4.0) |
| Ku | POL | 52.7 (±7.3) |
| $Ku_{min}$ | POL | 774 (±41.7) |
| $Ku_{core}$ | POL | 291 (±88.3) |
| Ku | LIG | 81.4 (±3.9) |
| $Ku_{min}$ | LIG | 571 (±26.6) |
| $Ku_{core}$ | LIG | 274 (±7.8) |
| Ku D247A | LigD | 189 (±4.6) |
| Ku D250A | LigD | 219 (±8.1) |
| Ku L251A | LigD | 134 (±5.1) |
| Ku S258A | LigD | 258 (±5.0) |
| Ku R262A | LigD | 125 (±4.7) |

**Supplementary Table S10:** LigD-DNA Binding Affinity Apparent $K_D$ Values

| DNA Size (bp) | $K_D$±SD (μM) |
|---|---|
| 10 | 1.10 (±0.07) |
| 15 | 0.38 (±0.02) |
| 20 | 0.35 (±0.02) |
| 30 | 0.43 (±0.03) |
| 40 | 0.35 (±0.03) |

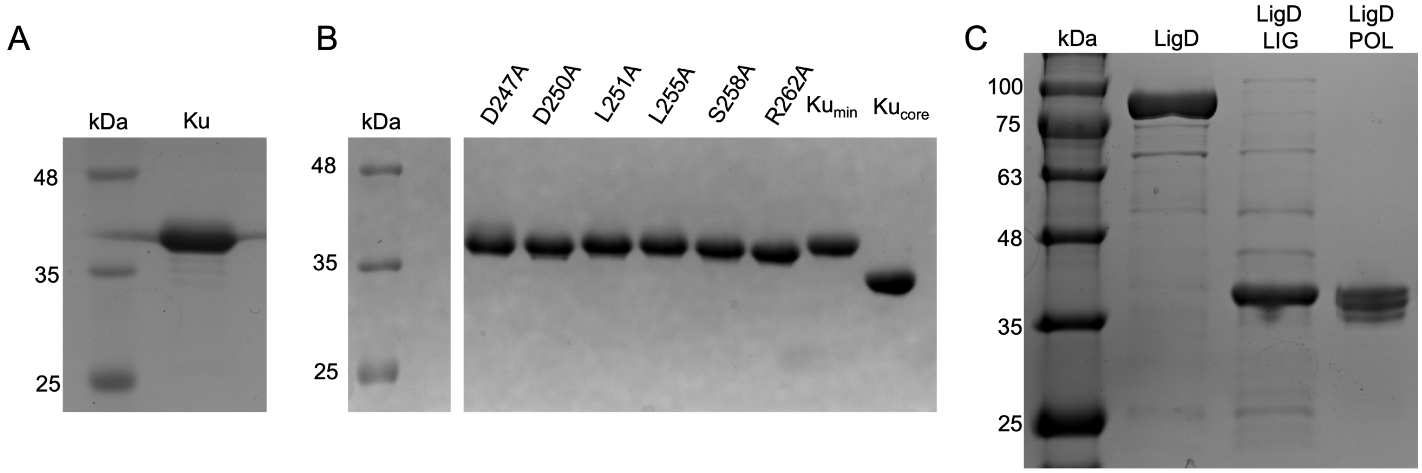


**Supplementary Figure S1:** Purified proteins used throughout this study. (A) $Ku_{WT}$ (B) Ku point mutants, $Ku_{min}$ and $Ku_{core}$ (C) LigD, LIG, and POL. 2μg of each purified protein was loaded onto a 12% SDS-PAGE gel, run at 180V for 60 minutes.

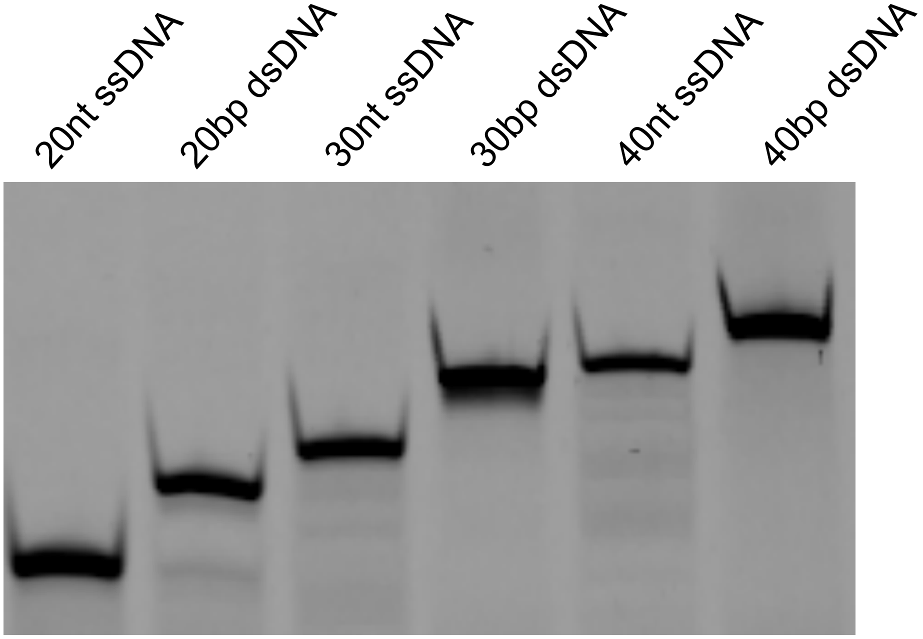


**Supplementary Figure S2:** Annealed DNA substrates used in this study.10 nM of each substrate was loaded after annealing for dsDNA substrates, as described in the methods. Electrophoresis was conducted on a 4-20% non-denaturing PAGE gel at 180V for 30 minutes in 0.5X TBE running buffer. Products were visualized using the Amersham Typhoon imager (GE Healthcare).

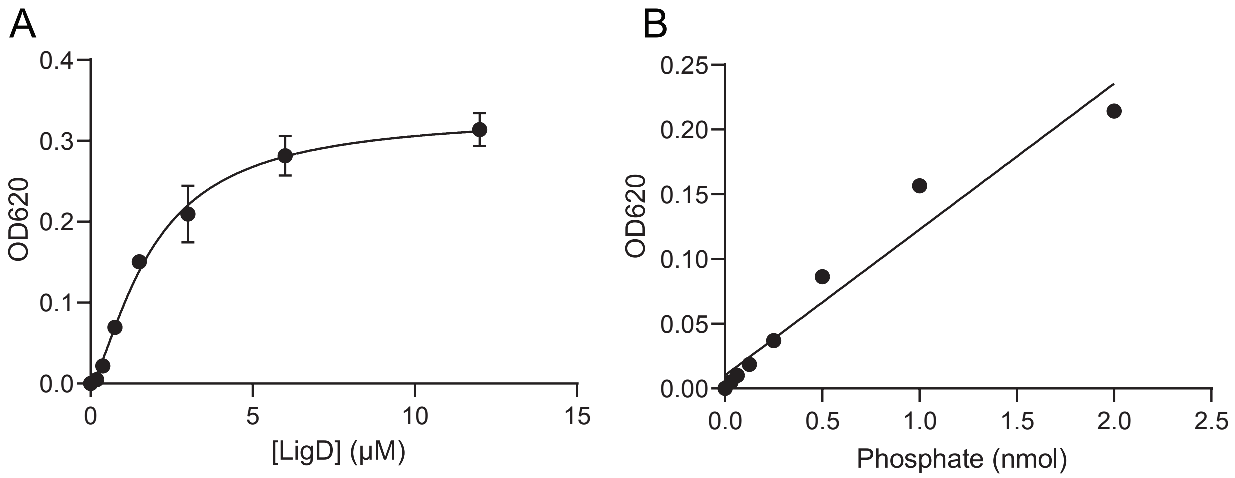


**Supplementary Figure S3:** Biomol green standards for phosphate released by LigD ligation. (A) $OD_{620nm}$ vs. 2-fold serial dilutions of LigD to determine that LigD is the rate limiting step in the Biomol green assay, as described in the methods. Ligation reactions were incubated at 37˚C for 30 minutes and then left to react with Biomol green solution for 5 minutes prior to imaging on a Biotek Synergy Neo2 plate reader. (B) Standard curve for the $OD_{620nm}$ vs. phosphate standards [$PO^{4-}$], to measure the release of phosphate using Biomol green.

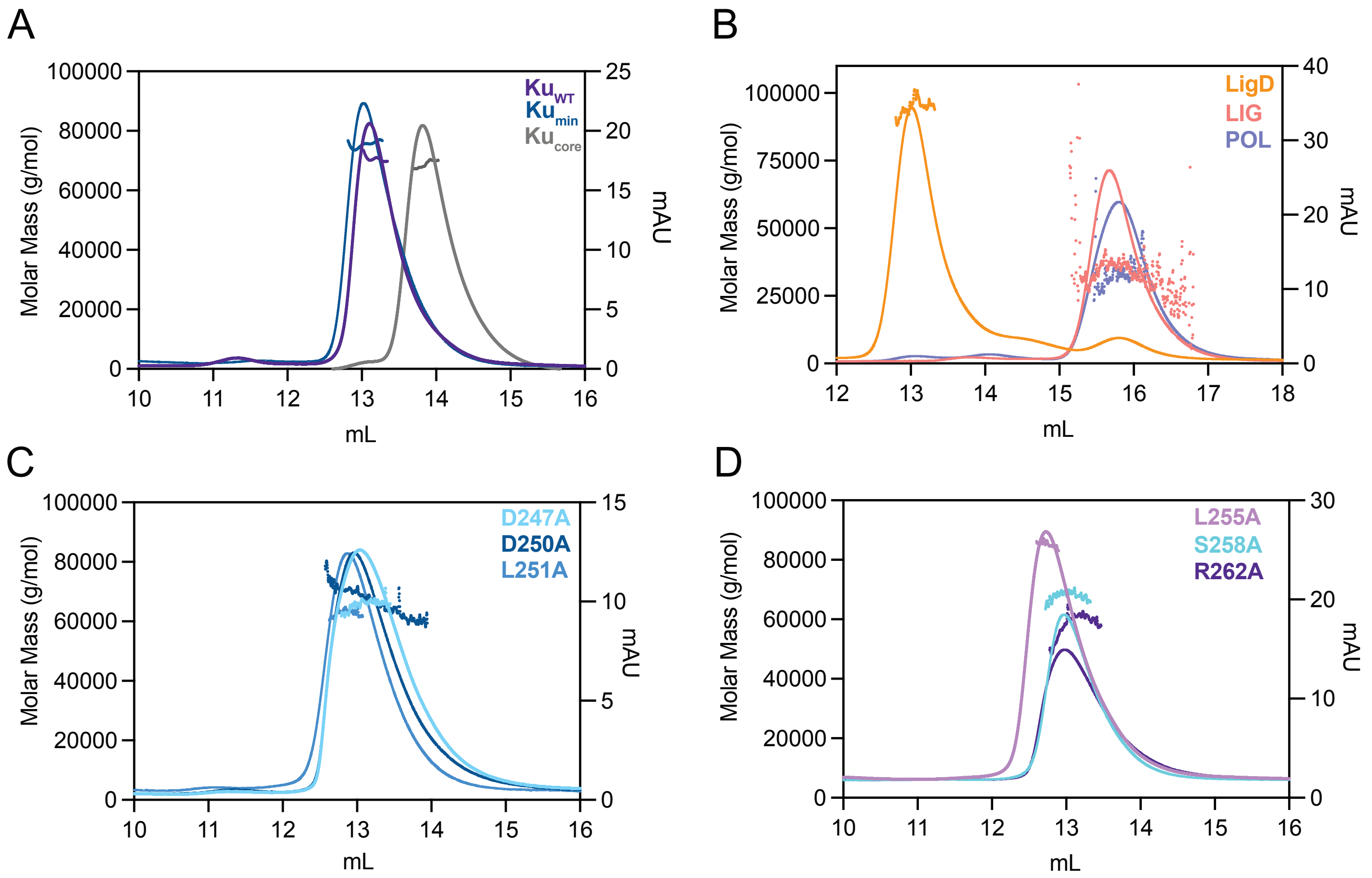


**Supplementary Figure S4:** Elution profiles (mAU) and molar mass (g/mol) from size-exclusion chromatography coupled to multi-angle light scattering (SEC-MALS) for purified proteins used throughout this study. SEC-MALS data for 50 µM of (A) $Ku_{WT}$, $Ku_{min}$, and $Ku_{core}$ (B) LigD, ligase domain of LigD (LIG), polymerase domain of LigD (POL), (C) Ku D247A, Ku D250A, Ku L251A (D) Ku L255A, Ku S258A and Ku R262A. Elution volume is represented by the elution peaks, while molar mass is denoted as the line of best fit.

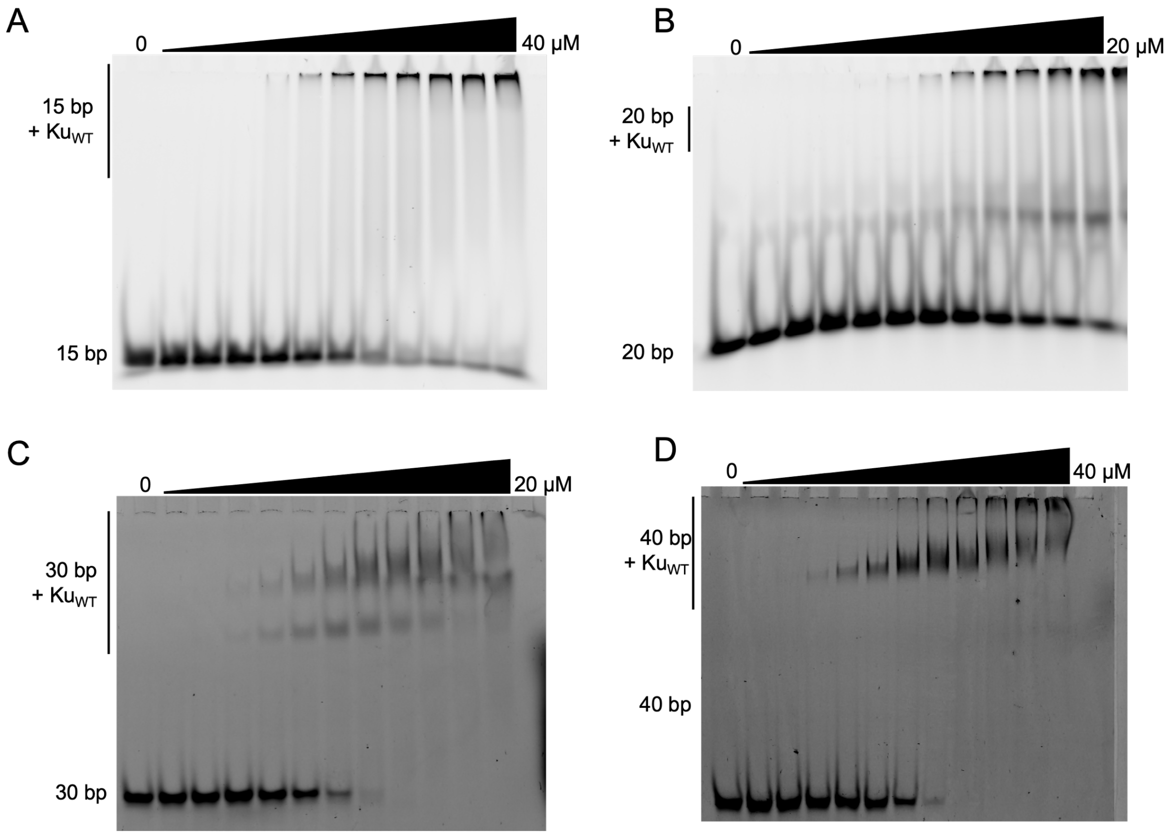


**Supplementary Figure S5:** Representative gel images for DNA binding shown in Figure 2A for Ku$_{WT}$ binding (A) 15 bp, (B) 20 bp, (C) 30 bp and (D) 40 bp. Samples were incubated at 30˚C for 20 minutes prior to loading on an 8% (A,B) or 4-20% (C,D) non-denaturing PAGE. Electrophoresis was conducted at 180V for 30 minutes in 0.5X TBE running buffer. Products were visualized using the Amersham Typhoon imager (GE Healthcare).

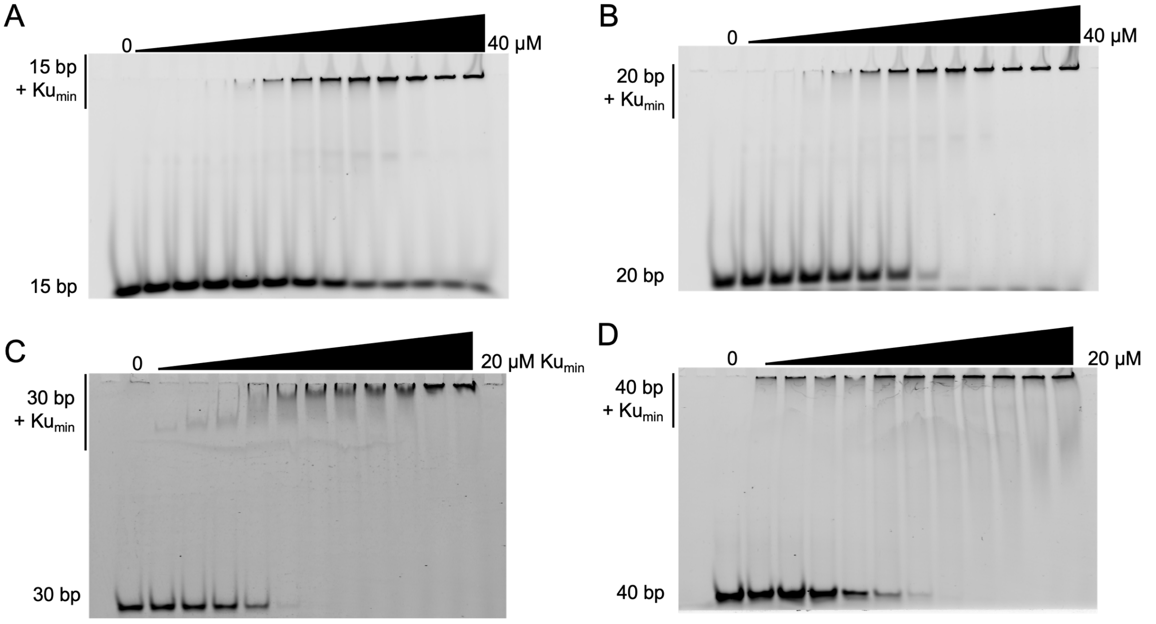


**Supplementary Figure S6:** Representative gel images for DNA binding shown in Figure 2C for Ku$_{min}$ binding (A) 15 bp, (B) 20 bp, (C) 30 bp and (D) 40 bp. Samples were incubated at 30˚C for 20 minutes prior to loading on an 8% (A,B) or 4-20% (C,D) non-denaturing PAGE. Electrophoresis was conducted at 180V for 30 minutes in 0.5X TBE running buffer. Products were visualized using the Amersham Typhoon imager (GE Healthcare).

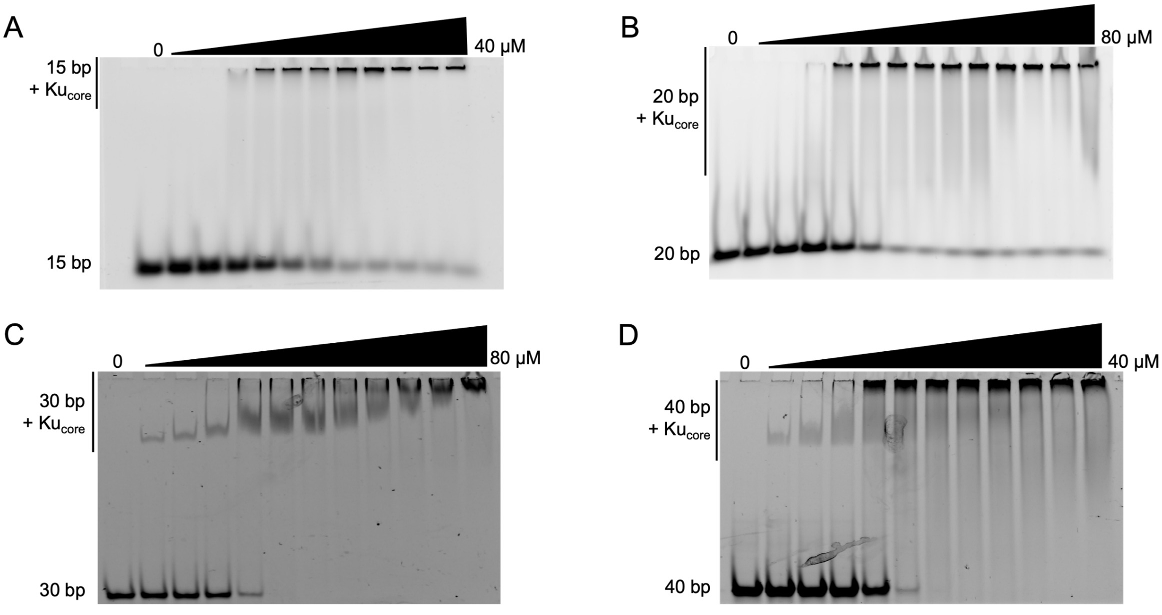


**Supplementary Figure S7:** Representative gel images for DNA binding shown in Figure 2E for Ku$_{core}$ binding (A) 15 bp, (B) 20 bp, (C) 30 bp and (D) 40 bp. Samples were incubated at 30˚C for 20 minutes prior to loading on an 8% (A,B) or 4-20% (C,D) non-denaturing PAGE. Electrophoresis was conducted at 180V for 30 minutes in 0.5X TBE

running buffer. Products were visualized using the Amersham Typhoon imager (GE Healthcare).

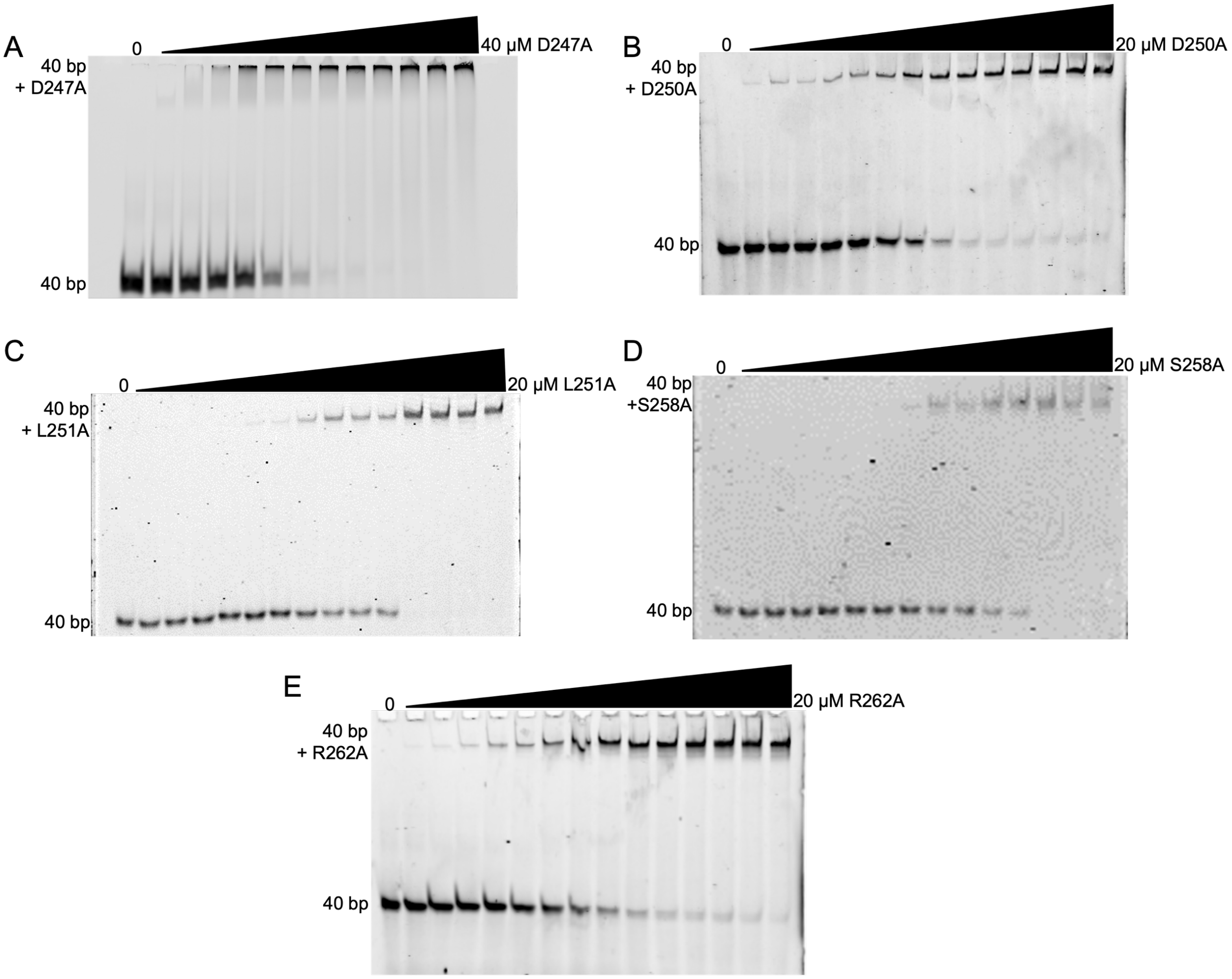


**Supplementary Figure S8:** Representative gel images for DNA binding shown in Figure 3A-B for Ku mutants (A) D247A, (B) D250A, (C) L251A, (D) S258A, and (E) R262A binding to a 40 bp DNA substrate. Samples were incubated at 30˚C for 20 minutes prior to loading on an 8% non-denaturing PAGE. Electrophoresis was conducted at 180V for 30 minutes in 0.5X TBE running buffer. Products were visualized using the Amersham Typhoon imager (GE Healthcare).

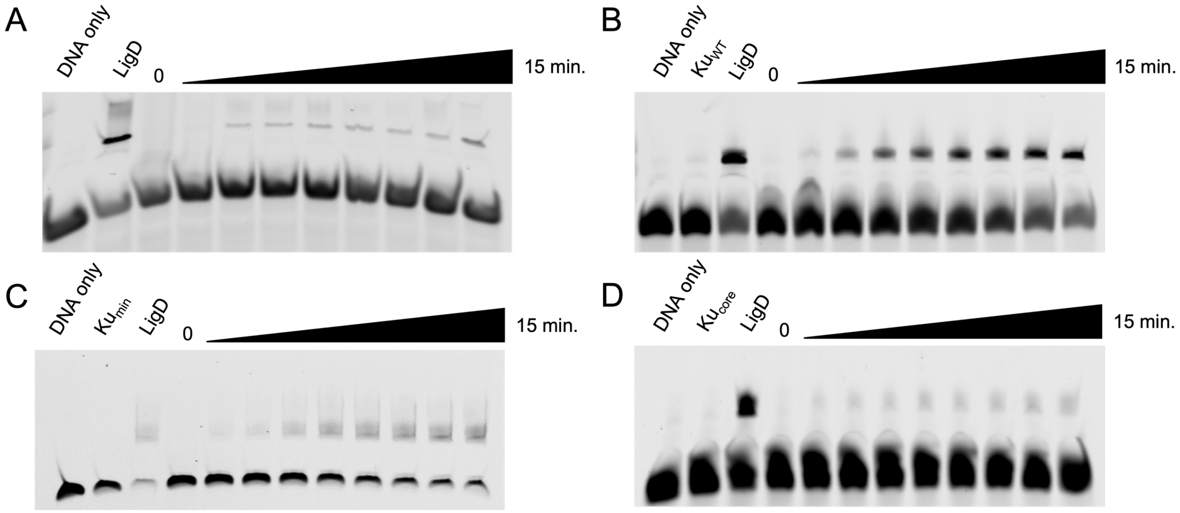


**Supplementary Figure S9:** Representative gel images for nick-sealing ligation in Figure 6 A, B by (A) 0.5 μM LigD (B) 1μM $Ku_{WT}$ with 0.5 μM LigD, (C) 1μM $Ku_{min}$ with 0.5 μM LigD, and (D) 1μM $Ku_{core}$ with 0.5 μM LigD. Reactions were incubated at 37˚C and quenched at the following time intervals: 0, 0.5, 1, 2.5, 5, 7.5, 10, 12.5 and 15 minutes, prior to loading on a 20% denaturing PAGE. Electrophoresis was conducted at 200V for 90 minutes in 1X TBE running buffer. Products were visualized using the Amersham Typhoon imager (GE Healthcare).

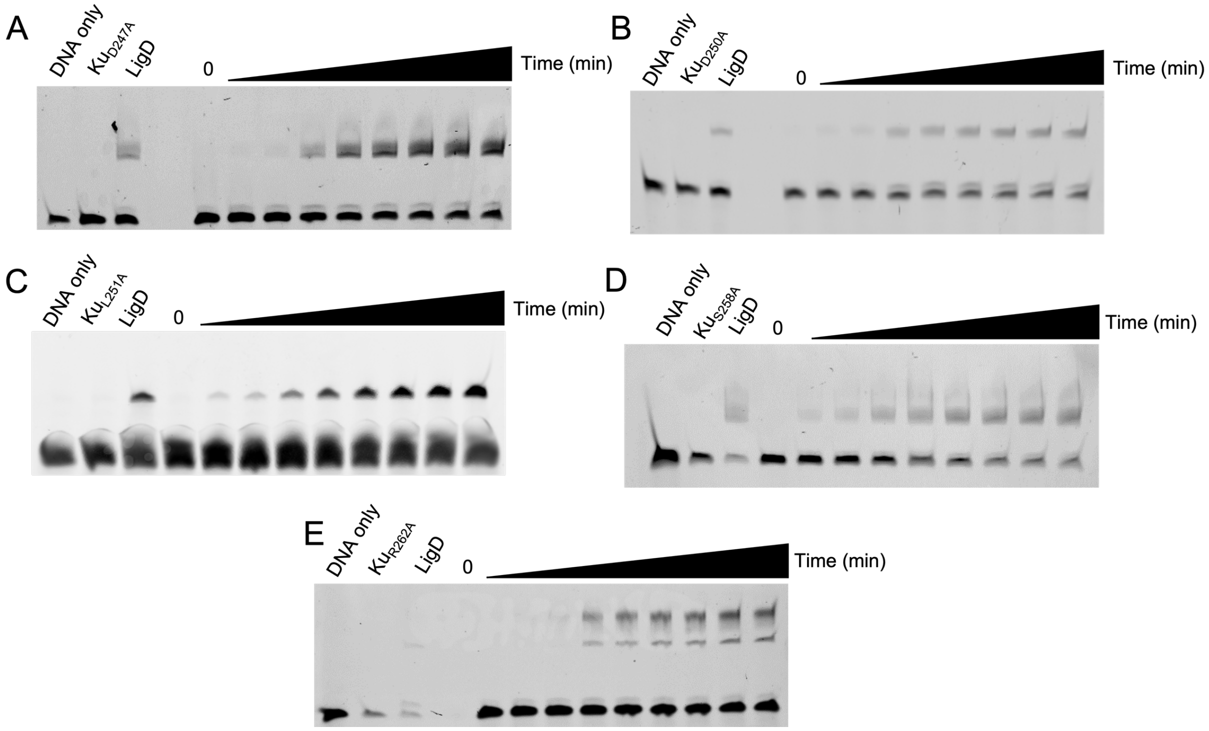


**Supplementary Figure S10:** Representative gel images for nick-sealing ligation in Figure 6 C, D by 1μM of each Ku mutants (A) D247A with 0.5 μM LigD (B) D250A with 0.5 μM LigD, (C) L251A with 0.5 μM LigD, (D) S258A with 0.5 μM LigD, and (E) R262A with 0.5 μM LigD. Reactions were conducted at 37˚C and were quenched at the following time intervals: 0, 0.5, 1, 2.5, 5, 7.5, 10, 12.5 and 15 minutes, prior to loading on a 20% denaturing PAGE. Electrophoresis was conducted at 200V for 90 minutes in 1X TBE running buffer. Products were visualized using the Amersham Typhoon imager (GE Healthcare).

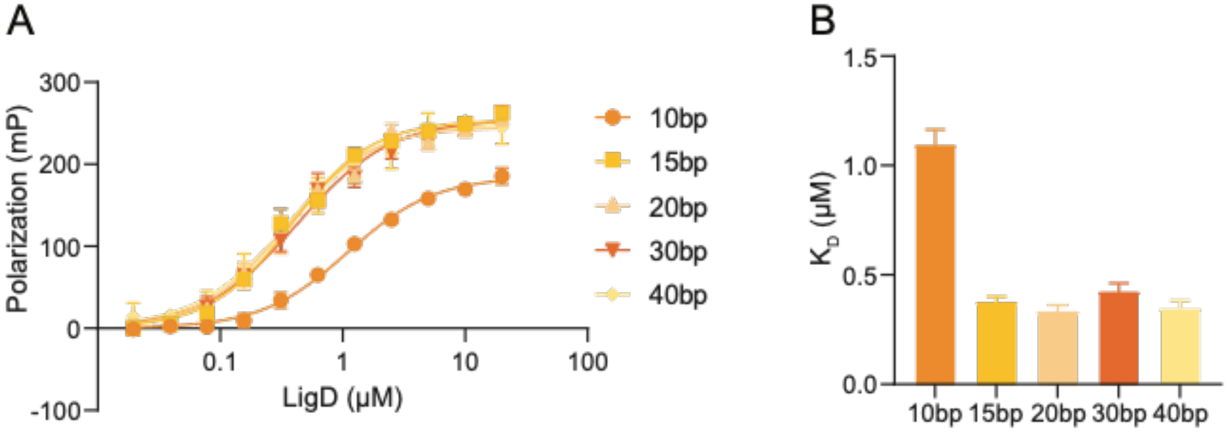


**Supplementary Figure S11:** LigD-DNA binding as measured by fluorescence polarization (FP). Reactions for FP were incubated in a 384-well plate at 30˚C for 20 minutes before imaging on a Biotek Synergy Neo2 plate reader. (A) Change in polarization plotted as a function of LigD concentration with binding to 10 bp, 15 bp, 20 bp, 30 bp, and 40bp. (B) Apparent $K_D$ for LigD binding to 10 bp, 15 bp, 20 bp, 30 bp, and 40bp. Data are plotted as the mean ± SD for n=3 technical replicates.

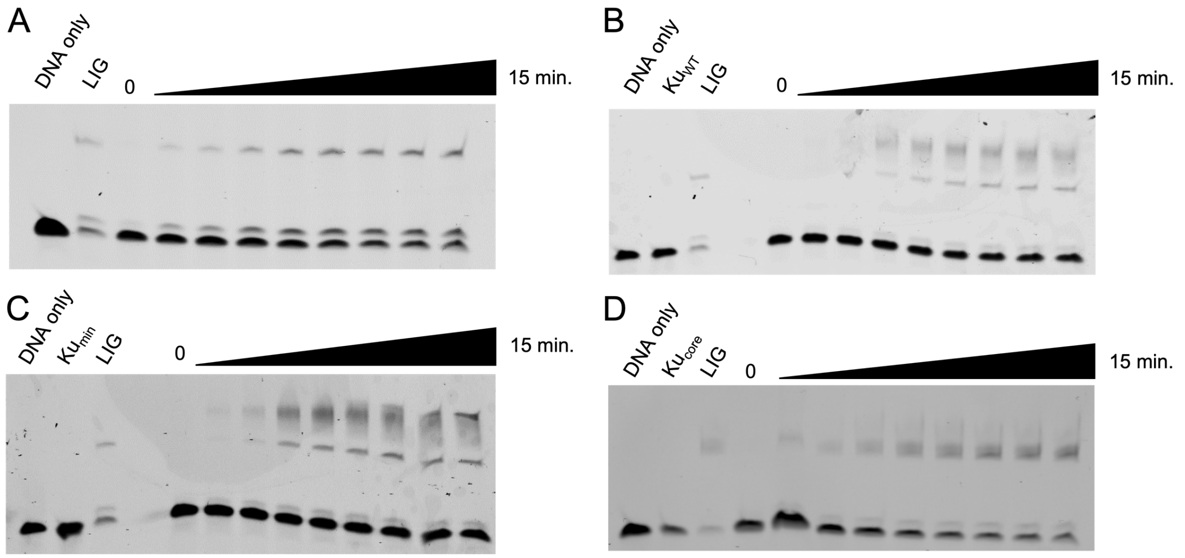


**Supplementary Figure S12:** Representative gel images for nick-sealing ligation in Figure 10 by (A) 0.5 µM LIG (B) 1µM $Ku_{WT}$ with 0.5 µM LIG, (C) 1µM $Ku_{min}$ with 0.5 µM LIG, and (D) 1µM $Ku_{core}$ with 0.5 µM LIG. Reactions were incubated at 37˚C and quenched at the following time intervals: 0, 0.5, 1, 2.5, 5, 7.5, 10, 12.5 and 15 minutes, prior to loading on a 20% denaturing PAGE. Electrophoresis was conducted at 200V for 90 minutes in 1X TBE running buffer. Products were visualized using the Amersham Typhoon imager (GE Healthcare).

## Supplementary References


1. Eschenfeldt,W.H., Stols,L., Sanville Millard,C., Joachimiak,A. and Donnelly,M.I. (2009) A family of LIC vectors for high-throughput cloning and purification of proteins. *Methods Mol Biol*, **498**, 105–115.
2. Zheng,L., Baumann,U. and Reymond,J.-L.L. (2004) An efficient one-step site-directed and site-saturation mutagenesis protocol. *Nucleic Acids Res.*, **32**, e115.
